# Genomic characterization of colorectal tumors: insights into significantly mutated genes, pathways, and survival outcomes

**DOI:** 10.1186/s12885-025-15440-x

**Published:** 2025-12-18

**Authors:** Tabitha A. Harrison, Syed H. Zaidi, Hang Yin, Robert S. Steinfelder, Conghui Qu, Elom K. Aglago, Sonja I. Berndt, Lisa A. Boardman, Hermann Brenner, Daniel D. Buchanan, Peter T. Campbell, Yin Cao, Andrew T. Chan, Stephen J. Chanock, Kimberly F. Doheny, David A. Drew, Jane C. Figueiredo, Amy J. French, Steven Gallinger, Peter Georgeson, Marios Giannakis, Ellen L. Goode, Stephen B. Gruber, Andrea Gsur, Marc J. Gunter, Sophia Harlid, Michael Hoffmeister, Wen-Yi Huang, Meredith AJ. Hullar, Jeroen R. Huyghe, Mark A. Jenkins, Yi Lin, Victor Moreno, Neil Murphy, Polly A. Newcomb, Christina C. Newton, Jonathan A. Nowak, Mireia Obón-Santacana, Shuji Ogino, Tameka Shelford, Mingyang Song, Claire E. Thomas, Amanda E. Toland, Tomotaka Ugai, Caroline Y. Um, Bethany Van Guelpen, Quang M. Trinh, Wei Sun, Thomas J. Hudson, Li Hsu, Ulrike Peters, Amanda I. Phipps

**Affiliations:** 1 Public Health Sciences Division, Fred Hutchinson Cancer Center, Seattle, WA USA; 2 Department of Epidemiology, School of Public Health, University of Washington, Seattle, WA USA; 3 Ontario Institute for Cancer Research, Toronto, ON Canada; 4 Institute for Public Health Genetics, School of Public Health, University of Washington, Seattle, WA USA; 5 Department of Epidemiology and Biostatistics, School of Public Health, Imperial College London, London, UK; 6 Division of Cancer Epidemiology and Genetics, National Cancer Institute, National Institutes of Health, Bethesda, MD USA; 7 Comprehensive Cancer Center, Mayo Clinic, Rochester, MN USA; 8 Division of Clinical Epidemiology and Aging Research, German Cancer Research Center (DKFZ), Heidelberg, Germany; 9 Division of Preventive Oncology, German Cancer Research Center (DKFZ) and National Center for Tumor Diseases (NCT), Heidelberg, Germany; 10 Colorectal Oncogenomics Group, Department of Clinical Pathology, Melbourne Medical School, The University of Melbourne, Parkville, Australia; 11 University of Melbourne Centre for Cancer Research, The University of Melbourne, Parkville, Australia; 12 Genomic Medicine and Family Cancer Clinic, The Royal Melbourne Hospital, Parkville, Victoria Australia; 13 Department of Epidemiology and Population Health, Albert Einstein College of Medicine, Bronx, NY USA; 14 Department of Surgery, Division of Public Health Sciences, Washington University School of Medicine, St Louis, MO USA; 15 Alvin J. Siteman Cancer Center at Barnes-Jewish Hospital and Washington University School of Medicine, St Louis, MO USA; 16 Division of Gastroenterology, Massachusetts General Hospital, Harvard Medical School, Boston, MA USA; 17 Channing Division of Network Medicine, Brigham and Women’s Hospital, Harvard Medical School, Boston, MA USA; 18 Clinical and Translational Epidemiology Unit, Massachusetts General Hospital, Harvard Medical School, Boston, MA USA; 19 Center for Inherited Disease Research (CIDR), Department of Genetic Medicine, Johns Hopkins University School of Medicine, Baltimore, MD USA; 20 Department of Medicine, Samuel Oschin Comprehensive Cancer Institute, Cedars-Sinai Medical Center, Los Angeles, CA USA; 21 Department of Population and Public Health Sciences, Keck School of Medicine, University of Southern California, Los Angeles, CA USA; 22 Department of Laboratory Medicine and Pathology, Division of Laboratory Genetics, Mayo Clinic, Rochester, MN USA; 23 Lunenfeld Tanenbaum Research Institute, Mount Sinai Hospital, University of Toronto, Toronto, ON Canada; 24 Department of Medical Oncology, Dana-Farber Cancer Institute, Boston, MA USA; 25 Broad Institute of MIT and Harvard, Cambridge, MA USA; 26 Department of Medicine, Brigham and Women’s Hospital, Harvard Medical School, Boston, MA USA; 27 Department of Quantitative Health Sciences, Division of Epidemiology, Mayo Clinic, Rochester, MN USA; 28 Department of Medical Oncology and Therapeutics Research and Center for Precision Medicine, City of Hope National Medical Center, Duarte, CA USA; 29 Center for Cancer Research, Medical University of Vienna, Vienna, Austria; 30 Nutrition and Metabolism Branch, International Agency for Research on Cancer, World Health Organization, Lyon, France; 31 Department of Diagnostics and Intervention, Oncology Unit, Umeå University, Umeå, Sweden; 32 Centre for Epidemiology and Biostatistics, Melbourne School of Population and Global Health, The University of Melbourne, Melbourne, Victoria Australia; 33 Oncology Data Analytics Program (ODAP), Unit of Biomarkers and Suceptibility (UBS), Catalan Institute of Oncology (ICO), L’Hospitalet de Llobregat, Barcelona, Spain; 34 ONCOBELL Program, Bellvitge Biomedical Research Institute (IDIBELL), L’Hospitalet de Llobregat, Barcelona, Spain; 35 Consortium for Biomedical Research in Epidemiology and Public Health (CIBERESP), Madrid, Spain; 36 Department of Population Science, American Cancer Society, Atlanta, GA USA; 37 Program in MPE Molecular Pathological Epidemiology, Department of Pathology, Brigham and Women’s Hospital, Harvard Medical School, Boston, MA USA; 38 Department of Pathology, Brigham and Women’s Hospital, Harvard Medical School, Boston, MA USA; 39 Department of Epidemiology, Harvard T.H. Chan School of Public Health, Harvard University, Boston, MA USA; 40 Cancer Immunology Program, Dana-Farber Cancer Institute, Boston, MA USA; 41 Division of Nutrition, Harvard Medical School, Boston, MA USA; 42 Departments of Epidemiology and Nutrition, Harvard T.H. Chan School of Public Health, Harvard University, Boston, MA USA; 43 Clinical and Translational Epidemiology Unit, Division of Gastroenterology, Massachusetts General Hospital, Harvard Medical School, Boston, MA USA; 44 Departments of Cancer Biology and Genetics and Internal Medicine, Comprehensive Cancer Center, The Ohio State University, Columbus, OH USA; 45 Wallenberg Centre for Molecular Medicine, Umeå University, Umeå, Sweden; 46 Department of Biostatistics, School of Public Health, University of Washington, Seattle, WA USA

**Keywords:** Colorectal neoplasm, Survival, Somatic mutations, Targeted sequencing

## Abstract

**Background:**

Identifying significantly mutated genes in tumors aids in understanding disease etiology and survival and may aid in the discovery of new drug targets. We aimed to detect and characterize mutated genes from a large, well-characterized group of colorectal cancers.

**Methods:**

In tumor and paired normal samples from 6,111 colorectal patients, we sequenced 199 genes identified from whole exome sequencing of over 1,100 tumors. Analyses focused on non-silent mutations. We classified significantly mutated genes after stratification by hypermutation status, and estimated associations of mutated genes/pathways with disease-specific (DS)-survival using Cox regression, adjusting for age, sex, mutation burden, hypermutation status, and study while accounting for multiple comparisons (*n* = 4,874).

**Results:**

We identified 57 genes that were significantly mutated in colorectal cancer, including 9 that were not previously reported. Among individual genes, only *BRAF* p.V600E mutations were significantly associated with poorer survival after correction for multiple testing (HR 1.96, *P* = 2.07 × 10^− 10^), with a more pronounced association among those with non-hypermutated tumors (HR 2.24, *P* = 1.79 × 10^− 12^). We also observed statistically significant associations with survival for four mutated pathways: TP53/ATM (HR 1.24, *P* = 7.96 × 10^− 4^), RTK/RAS (HR 1.33, *P* = 3.81 × 10^− 6^), TGF-beta (HR 1.25, *P* = 1.85 × 10^− 3^), and WNT (HR 0.81, *P* = 2.52 × 10^− 03^).

**Conclusions:**

We identified 9 significantly mutated genes, some of which are known drug targets. Among individual genes, only the *BRAF* p.V600E mutation was significantly associated with DS-survival, suggesting a limited survival impact from mutations driving colorectal cancer development.

**Supplementary Information:**

The online version contains supplementary material available at 10.1186/s12885-025-15440-x.

## Background

Colorectal cancer, a leading cause of cancer-related mortality worldwide, is a heterogeneous disease that evolves through multiple pathways defined by genomic events [1, 2]. Whole exome and genome sequencing studies have identified several key driver genes, such as *APC*, *TP53*, *KRAS*, *PIK3CA*, and *SMAD4*, providing insights into tumor development, novel pathways, and potential drug targets. To gain a more comprehensive understanding of the somatic-mutated genes significantly associated with colorectal cancer, including those that occur less frequently, large sample sizes in conjunction with sequencing approaches tailored to colorectal cancer are needed. Furthermore, knowledge about the variation in somatic mutation prevalence across key patient and tumor characteristics, such as sex, tumor stage, and anatomical site, remains limited, as sizable studies are often based on data from tertiary treatment centers or commercial sequencing efforts that primarily include late-stage cancers [3, 4]. Many studies support hypotheses about differing colorectal cancer etiologies and prognoses according to clinicopathological factors, including tumor location [5–7]. Reducing knowledge gaps in this area will be critical for better understanding disease etiology, tailoring personalized treatment strategies, and improving survival outcomes.

Identifying associations between somatic mutations and survival is pivotal for the development of prognostic biomarkers. Previously, studies into associations between mutated genes and colorectal cancer-specific survival or overall survival have yielded conflicting results, partially due to inadequate adjustment for known survival-related factors, such as tumor mutational burden and microsatellite instability (MSI), limited adjustment for multiple comparisons, and/or limited follow-up data to define colorectal cancer-specific survival [8, 9]. Addressing these factors necessitates larger sample sizes and rigorous statistical analyses to help explain existing inconsistencies.

To investigate novel somatic driver genes associated with colorectal cancer, somatic heterogeneity by patient and tumor characteristics, and somatic features associated with survival, we combined data from several well-characterized, population-based colorectal cancer studies and conducted centralized deep targeted sequencing of tumor and normal DNA to identify mutated genes at low variant allele frequencies. Sequencing panel genes were selected primarily from whole exome sequencing data of paired tumor-normal samples from 1,211 colorectal cancer patients in order to enable a well-powered study to identify novel genes in colorectal cancers that are significantly mutated above the background mutation rate [10]. In this study, colorectal cancer cases (*n* = 6,111) were collected from several population-based case-control studies and cohorts that included all stages of disease. A large fraction of the patients was followed up to assess outcome data, enabling analysis of colorectal cancer-specific death.

## Methods

### Study populations and sample processing

**Fig. 1:**
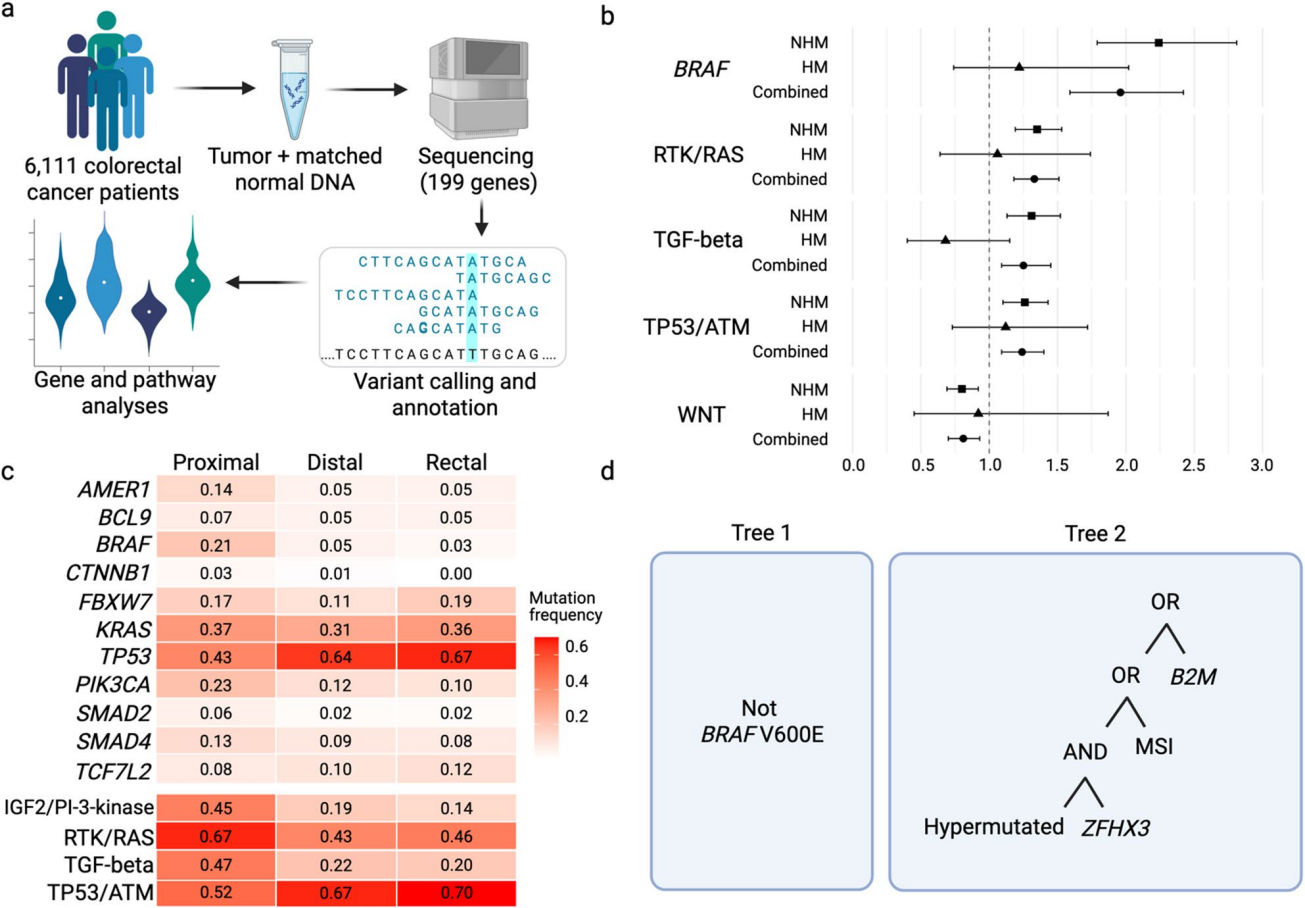
**a** Overview of study approach. **b** Forest plot of multivariate Cox proportional hazards regression analysis of disease-specific death for significant gene and pathway findings. Significance threshold was determined based on Bonferroni correction for combined samples (171 genes, p value < 2.9 × 10^− 4^ and 6 pathways, p value < 8.3 × 10^− 3^). Hazard ratios (HR) and 95% confidence intervals (CI) are shown for combined and stratified (non-hypermutated, NHM, and hypermutated, HM) analyses. **c** Heat map showing mutation frequencies for genes and pathways with significant mutation prevalence differences by tumor site (proximal colon, distal colon, rectal cancers). **d** Boolean tree combinations from logic regression analysis of colorectal tumor features in relation to disease-specific survival. Survival time was transformed using the cumulative hazard function. Features included mutated gene, hypermutation status, MSI-status, and mutational signatures. Tree 1 = Non-mutated *BRAF*. Tree 2 = (Hypermutated AND mutated *ZFHX3*) OR MSI-H OR mutated *B2M*

We sequenced tumor and matched normal DNA samples and called somatic mutations for 6,111 colorectal cancer cases within 10 studies from the Genetics and Epidemiology of Colorectal Cancer Consortium (GECCO) and three sites from the Colon Cancer Family Registry (CCFR) (Fig. 1a, Supplementary Information). All patients provided written informed consent for the collection of specimens and data used in the analyses. The Ontario Institute for Cancer Research (OICR) and Center for Inherited Disease Research (CIDR) processed all the DNA samples. Sample processing, sequencing, and mutation calling for 2,542 samples sequenced at OICR has been described previously [11]. Here, we describe methods for 3,569 samples processed at CIDR. The analyses in this paper focus on 199 genes present on both the OICR and CIDR sequencing panels.

We completed tumor pathology reviews to confirm primary colorectal carcinoma and dissected tumor tissues from slides guided by hematoxylin and eosin-stained slides marked for tumor regions. We extracted DNA from formalin-fixed paraffin-embedded (FFPE) or fresh frozen tumor tissue and matched germline samples (primarily blood-derived, or normal mucosa or buccal in a small subset) using QIAamp DNA Mini or FFPE tissue kits (Qiagen, Inc.).

### Targeted sequencing

Panel content selection was primarily based on analyses of 1,211 whole exome sequenced, paired normal-tumor samples from colorectal cancer patients in The Cancer Genome Atlas (TCGA) and two prospective cohort studies (Supplementary Information) [10]. For this analysis, we performed targeted, paired-end sequencing using a custom Integrated DNA Technologies (IDT) design with an xGen library prep and Illumina NovaSeq6000 (Illumina, Inc.). We sheared genomic DNA using the Covaris LE220plus instrument (Covaris, Inc.) and used Kapa Hyper prep kits to amplify dual-indexed adapter ligated fragments and GE Healthcare Sera-Mag Magnetic SpeedBeads (Carboxylate-Modified) for library clean up. We included 750ng of amplified library in a pooled enrichment reaction (eight samples per normal pool, four samples per tumor pool) following IDT protocols and performed post-capture amplification using the Kapa HiFi PCR enzyme with custom primers. See the Supplementary Information for details about genomic content, library preparation, and sequencing. The mean coverages for tumor and normal sequencing were 975x and 273x, respectively.

We aligned reads to the reference human genome GRCh37/hg19 using Burrows-Wheeler Aligner (BWA-MEM, version 0.7.17). We used the GRCh37/hg19 reference build for alignment and variant calling to maintain consistency with legacy data sources (e.g., TCGA) and previous colorectal cancer studies. We note that the targeted coding regions sequenced in this panel are highly conserved between GRCh37 and GRCh38, minimizing the impact of genome build on variant detection and interpretation. We called single nucleotide variants and indels by taking the intersection of mutation calls from Strelka2 (version 2.9.9) and MuTect2 (GATK version 4.1.4.1), and applying the following additional filters: strand bias, minor allele frequency in the Exome Aggregation Consortium (< 0.01%), variant allele frequency (*≥* 20%), and read position, aiding in minimization of false positives from low-purity or normal-contaminated samples [12, 13]. We annotated somatic mutation calls using ANNOVAR and identified significantly mutated genes using MutSigCV [14, 15]. We defined mutations and pathways using our previously described approach, defining specific mutations for a subset of well-described genes (*BRAF*: codon p.V600E mutations; *CTNNB1* hotspot; *KRAS*: codons G12, G13, Q61, K117, A146 mutations; *PIK3CA*: transcript NM181523; and *TP53*: transcript NM00546 encoding for the canonical p53) [11, 16]. We called microsatellite instability (MSI) using MSIsensor2 with a score of *≥* 18.0% as MSI-high (MSI-H), and empirically determined a mutation burden threshold to define hypermutation status, designating tumors as hypermutated if they had *≥* 26 somatic mutations (13 mutations per million bases) and non-hypermutated otherwise [11, 16]. See the Supplementary Information for additional details about somatic mutation calling, including filtering and post-mutation calling QC.

### Statistical analyses

We applied MutSigCV, utilizing a likelihood ratio test to identify genes significantly mutated beyond the background mutation rate and applying the Benjamini-Hochberg procedure to account for multiple testing, as is consistent with the literature and methods [14]. We used logistic regression, adjusting for age at diagnosis, sex, log-mutation burden, and MSI, to assess differences in mutational frequencies of genes and pathways by patient sex (female/male), tumor stage (stage I/II & III/IV), and tumor site (proximal/distal/rectal). These analyses were limited to genes with *≥* 10 mutated tumors in a stratum (*n* = 1 gene dropped from the 199 set). Therefore, we assessed statistical significance using Bonferroni p-value thresholds to account for multiple testing of 198 genes (2.5 × 10^− 4^) and six key colorectal cancer pathways (8.3 × 10^− 3^).

We performed Cox regression to estimate hazard ratios (HRs) and 95% confidence intervals (CIs) for associations of somatically mutated genes and pathways with disease-specific (DS)-survival, adjusting for age at diagnosis, sex, mutation burden (log of the total mutation number), and hypermutation status, stratifying baseline hazards by study population. We adjusted for mutational burden and hypermutation status, as we previously demonstrated that both impact survival outcomes [11]. In primary analyses, we did not adjust for stage at diagnosis. This choice reflects our expectation that stage most likely operates as a mediator in relationships of somatically mutated genes and/or pathways with DS-survival. That is, certain somatic mutations may be expected to impact DS-survival by contributing to more aggressive tumor biology, leading to later stage at diagnosis and subsequently poorer outcomes. Thus, adjusting for stage would be expected to mask part of the mechanism through which these somatic mutations contribute to outcomes. However, to provide further context and support comparability to some prior investigations, we also conducted secondary analyses including, stage adjustment, where observed findings reflect survival associations independent of impacts on stage at diagnosis. We evaluated violations of the proportional hazards assumption by testing for a nonzero slope of the scaled Schoenfeld residuals on the ranked failure time, and no violations were observed. Survival analyses were restricted to tests of genes for which *≥* 10 colorectal cancer deaths were observed among those with mutated tumors (*n* = 28 genes dropped). Therefore, we assessed statistical significance using Bonferroni p-value thresholds to account for multiple testing of 171 genes (2.9 × 10^− 4^) and six pathways (8.3 × 10^− 3^).

We performed logic regression to explore logic combinations of binary covariates in relation to DS-survival, using an exponential survival model by transforming survival time via the cumulative hazard function [17]. This approach is well-suited for identifying non-additive combinations of binary predictors, enabling interpretable modeling of combinations of genomic events. Given our focus on mutation co-occurrence and combinatorial effects, logic regression provides a biologically intuitive complement to additive multivariable models. The covariates included hypermutation status (hypermutated, non-hypermutated), MSI status (MSI-H, MSS), and mutation status for the 69 genes that were mutated in more than 5% of the samples (mutated, non-mutated). We adaptively selected logic combinations of model covariates using a simulated annealing algorithm and selected the best performing logic regression model using 5-fold cross-validation with random seeds. We expressed binary variable combinations in tree form using logic operators “AND” and “OR” and represented the regression model as a score function of combinations of multiple logic trees, where the score was the partial likelihood in a Cox proportional hazard regression model. We performed sub-analyses stratifying by hypermutation status.

## Results

The clinicopathologic characteristics of the 6,111 colorectal cancer patients are described in Table 1. The number of mutations per tumor ranged from zero to 1,128 (mean: 28.7 mutations). A total of 1,106 (18.1%) tumors were hypermutated and, of these, 961 (86.9%) were MSI-H and/or had missense mutations in the *POLE* exonuclease domain. We identified 39 unique missense mutations in the exonuclease domain of *POLE* (Supplementary Table S1). Among the recurrent *POLE* mutations detected in three or more tumors, the most frequent were p.Pro286Leu/Arg/Ser (*n* = 23), followed by p.Val411Leu (*n* = 11), p.Ser459Phe (*n* = 8), p.Ala456Pro (*n* = 6), and p.Phe367Cys/Ser/Val (*n* = 3). Four *POLE* exonuclease domain mutations (p.Lys398Met, p.His422Tyr, p.Thr449Arg, p.Arg375Gln) detected in single tumors were observed in non-hypermutated tumors. Mutations in a *POLD1* motif of highly conserved amino acids that bind magnesium (termed the DEDD motif and consisting of p.Asp316, p.Glu318, p.Asp402, and p.Asp515) disrupt the *POLD1* proofreading function [18]. We identified six tumors carrying missense mutations in the DEDD motif, all of which were hypermutated (Supplementary Table S2).

**Table 1:** Clinicopathologic characteristics for the complete dataset and for colorectal cancer deaths

	Overall (*N* = 6111)	CRC Death (*N* = 1185)
	*n* (%)	*n* (%)
Median Survival Time (Range), months	--	31.5 (17.9–50.2)
Median Age at Diagnosis (Range)	68 (20–93)	68 (21–92)
<50	690 (11.3%)	149 (12.6%)
50–65	1811 (29.6%)	352 (29.7%)
>65	3610 (59.1%)	684 (57.7%)
Sex		
Female	3344 (54.7%)	629 (53.1%)
Male	2767 (45.3%)	556 (46.9%)
Tumor stage		
Stage 1 or local	1190 (23.2%)	67 (6.4%)
Stage 2/3 or regional	3342 (65.2%)	582 (55.3%)
Stage 4 or distant	590 (11.5%)	403 (38.3%)
Missing	989	133
Tumor site		
Proximal	2497 (43.9%)	454 (40.3%)
Distal	1723 (30.3%)	345 (30.6%)
Rectal	1465 (25.8%)	327 (29.0%)
Missing	426	59
Study		
ACCFR	343 (5.6%)	55 (4.6%)
CORSA	174 (2.8%)	-
CPSII	541 (8.9%)	79 (6.7%)
CRA	330 (5.4%)	-
CRCGEN	796 (13.0%)	155 (13.1%)
DACHS	278 (4.5%)	48 (4.1%)
EPIC	193 (3.2%)	11 (0.9%)
HCCS	123 (2.0%)	-
HPFS	300 (4.9%)	89 (7.5%)
IWHS	393 (6.4%)	118 (10.0%)
MCCS	475 (7.8%)	161 (13.6%)
NHS	435 (7.1%)	118 (10.0%)
NHSII	45 (0.7%)	8 (0.7%)
OFCCR	683 (11.2%)	162 (13.7%)
PLCO	136 (2.2%)	30 (2.5%)
SCCFR	531 (8.7%)	151 (12.7%)
WHI	335 (5.5%)	-
MSI Status		
Stable	5122 (83.8%)	1104 (93.2%)
MSI-High	989 (16.2%)	81 (6.8%)
Hypermutation Status		
Non-hypermutated	5005 (81.9%)	1092 (92.2%)
Hypermutated	1106 (18.1%)	93 (7.8%)

*CRC* colorectal cancer, *MSI* microsatellite instability

### Significantly mutated genes

**Fig. 2:**
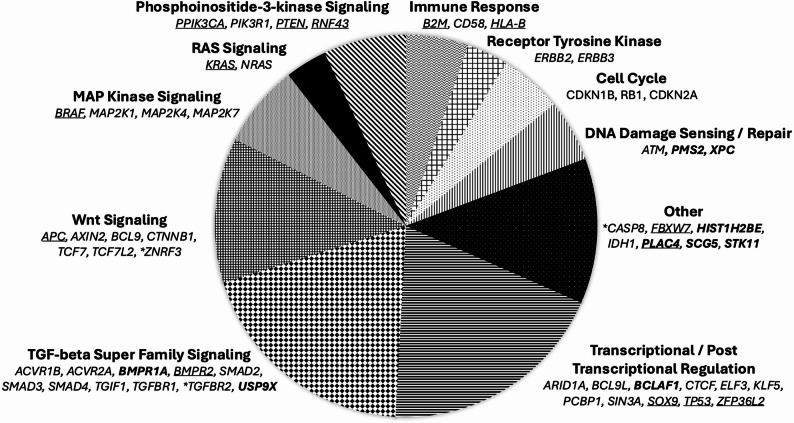
Somatically mutated genes that are significantly associated with colorectal cancer development. Bold marks 9 newly reported genes; genes marked with * are those only identified in hypermutated tumors at q < 10 × 10^− 05^; underlined genes are identified in both non-hypermutated tumors at q < 0.1 and hypermutated tumors at q < 10 × 10^− 05^; all other genes are identified in non-hypermutated tumors only at q < 0.1 based on MutSigCV. More detailed results are available in Supplementary Tables S3 and S4

We classified a total of 57 unique, significantly mutated genes across all tumors. Among non-hypermutated tumors, we classified 54 significantly mutated genes (q < 0.1), including 9 genes not previously reported as significantly mutated in colorectal tumors (Supplementary Table S3). Many of the genes are involved in well-known colorectal cancer pathways, such as the TGF-beta, WNT, MAPK, RAS or PI3K signaling pathways (Fig. 2). The 9 newly discovered genes included members of the TGF-beta signaling pathway (*BMPR1A*, *USP9X*), genes involved in DNA repair (*PMS2*, *XPC*), the chaperone involved in endoplasmic reticulum transport (*SCG5*), serine/threonine protein kinase (*STK11*), death-promoting transcriptional repressor (*BCLAF1)*, placenta specific protein (*PLAC4)*, and the core component of nucleosomes (*HIST1H2BE*). The identification of significantly mutated genes in hypermutated tumors is more difficult because of the overall high mutational burden. As such, more stringent thresholds were applied (q < 10^− 5^). This resulted in the identification of 17 significantly mutated genes, including 16 of which have been described previously as significantly mutated in colorectal cancer and *PLAC4*, which has not previously been described (Supplementary Table S4). Additional information about the mutations among hypermutated and non-hypermutated tumors for these significantly mutated genes, including in silico prediction metrics, is available in Supplementary Table S5 and Supplementary Figures S1 and S2.

### Patient and tumor characteristics

We identified statistically significant differences in the prevalence of certain somatic mutations by tumor site, tumor stage at diagnosis, and sex. With respect to tumor site, the mutation prevalence for the RTK/RAS, IGF2/PI-3-kinase, and TGF-beta (TGFB1) pathways was significantly lower in distal colon and rectal tumors than in proximal colon tumors, whereas the prevalence of *TP53*/*ATM* set mutations was significantly greater in distal colon and rectal tumors (Fig. 1c, Supplementary Table S6). Additionally, significant differences were noted by tumor site for the prevalence of mutations in the following genes: *KRAS*, *TP53*, *BRAF* p.V600E, *BCL9*, *AMER1*, *FBXW7*, *PIK3CA*, *TCF7L2*, *SMAD2*, *SMAD4*, and *CTNNB1*. Compared with stage 1 tumors, RTK/RAS pathway mutations occurred more frequently in stage 4 (OR 1.65, 95% CI 1.34–2.05, *P* = 3.86 × 10^− 06^), and stage 2 and 3 tumors (OR 1.32, 95% CI 1.14–1.52, *P* = 1.56 × 10^− 04^) (Supplementary Table S7). Furthermore, we observed statistically significant differences in the prevalence of *BRAF* p.V600E mutations in stage 4 versus stage 1 tumors (OR 3.43, 95% CI 2.31–5.09, *P* = 8.88 × 10^− 10^), and the prevalence of mutations in the IGF2/PI3K pathway was greater among stage 2 and 3 tumors than among stage 1 tumors (OR 1.29, 95% CI 1.08–1.55, *P* = 6.05 × 10^− 03^). Finally, mutations were less common in tumors among males than in those among females with respect to *MXRA5* (OR 0.60, 95% CI 0.47–0.77, *P* = 5.11 × 10^− 05^), *BRAF* p.V600E (OR 0.43, 95% CI 0.34–0.55, *P* = 2.57 × 10^− 11^), and the RTK/RAS pathway (OR 0.74, 95% CI 0.65–0.84, *P* = 1.61 × 10^− 06^) (Supplementary Table S8). Because MSI status is correlated with sex, tumor site, and tumor stage, we further explored analyses stratified by MSI status for the genes with significant differences in mutation prevalence by sex, site, and stage (Supplementary Tables S9-S11).

### Colorectal cancer disease-specific (DS)-survival

DS-survival outcome data were available for 4,874 colorectal cancer patients, of whom 1,185 patients (24.3%) died from colorectal cancer. The median survival time after diagnosis was 31.5 months (study-specific median times ranged from 17.9 to 50.2 months). DS-survival was significantly more favorable among individuals with hypermutated tumors than among those with non-hypermutated tumors (HR 0.42, 95% CI 0.31–0.58, *P* = 9.2 × 10^− 08^).

**Fig. 3:**
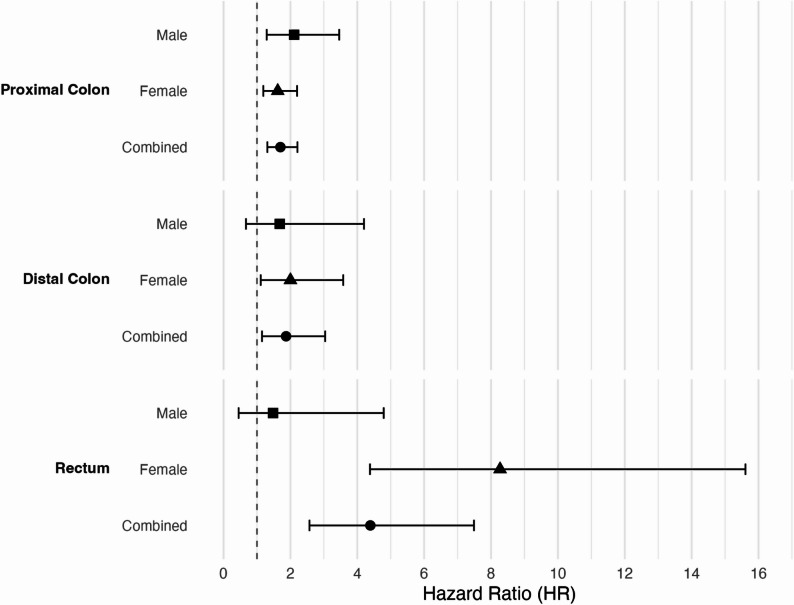
Forest plot of multivariate Cox proportional hazards regression analysis for disease-specific death. Hazard ratios (HR) and 95% confidence intervals (CI) are shown for the *BRAF* p.V600E mutation analyses stratified by tumor site and sex. More detailed results are available in Supplementary Table S13

In analysis of individual genes, tumors with *BRAF* p.V600E mutations were associated with worse DS-survival, after accounting for age, sex, log-mutation burden, and hypermutation status, and accounting from multiple comparisons (HR_All_ 1.96, 95% CI 1.59–2.42, *P* = 2.07 × 10^− 10^) (Fig. 1b, Supplementary Table 12). In analyses stratified by hypermutation status, the association with *BRAF* p.V600E mutation status was limited to cases with non-hypermutated tumors (HR_NHM_ 2.24, 95% CI 1.79–2.81, *P* = 1.79 × 10^− 12^, HR_HM_ 1.22, 95% CI 0.74–2.02, *P* = 0.44). Adjustment for stage resulted in more attenuated estimates, but the associations with survival and the pattern of association by hypermutation status remained the same (HR_All_ 1.70, 95% CI 1.35–2.13, *P* = 4.72 × 10^− 06^, HR_NHM_ 1.99, 95% CI 1.56–2.55, *P* = 4.12 × 10^− 08^, HR_HM_ 0.97, 95% CI 0.56–1.68, *P* = 0.91). As the frequency of *BRAF* p.V600E mutations differed significantly by tumor and patient characteristics, we further stratified DS-survival analyses by both tumor site and sex (Fig. 3, Supplementary Table S13). The presence of a *BRAF* p.V600E mutation was associated with poorer survival regardless of tumor site or sex but was most strongly associated with DS-survival for those with rectal tumors (HR 4.39, 95% CI 2.57–7.49, *P* = 5.94 × 10^− 08^) and particularly for women with rectal tumors (HR 8.27, 95% CI 4.38–15.61, *P* = 7.34 × 10^− 11^). However, these mutations were less common among those with rectal tumors (2.5% rectal tumors vs. 21.3% proximal tumors) (Supplementary Table S6), such that the number of mutations was small in some strata. Therefore, these sex- and tumor site-stratified findings should be interpreted with caution and are considered hypothesis generating. Replication in larger, independent cohorts will be needed to confirm these subgroup-specific associations.

Analyses of other somatically mutated genes in relation to DS-survival did not yield statistically significant associations after accounting for multiple comparisons, although we observed suggestive evidence for associations (*p* < 0.05) with *B2M* somatic mutations in all tumors (HR_All_ 0.52, 95% CI 0.34–0.82, *P* = 4.4 × 10^− 03^), *SMAD4* mutations in non-hypermutated tumors (HR_NMH_ 1.33, 95% CI 1.10–1.60, *P* = 3.2 × 10^− 03^), and *TP53* mutations in non-hypermutated tumors (HR_NHM_ 1.25, 95% CI 1.10–1.42, *P* = 5.7 × 10^− 04^) (Supplementary Table S12).

In pathway-level analyses, we observed statistically significant associations after accounting for multiple comparisons between DS-survival and mutations in RTK/RAS (HR_All_ 1.33, 95% CI 1.18–1.51, 3.81 × 10^− 06^), TP53/ATM (HR_All_ 1.24, 95% CI 1.09–1.40, 7.96 × 10^− 04^), TGF-beta (HR_All_ 1.25, 95% CI 1.09–1.45, 1.85 × 10^− 03^), and WNT (HR_All_ 0.81, 95% CI 0.70–0.93, 2.52 × 10^− 03^) (Supplementary Table S12). To assess whether pathway findings were driven primarily by a single gene, we further adjusted the analyses for the most significantly associated gene in each pathway. The findings for RTK/RAS were driven primarily by mutations in *BRAF* p.V600E, and the findings for TP53/ATM were driven primarily by mutations in *TP53*. The observed association between a mutated WNT pathway and DS-survival was attenuated but still present after adjusting for *APC* (HR_All_ 0.78, 95% CI 0.63–0.96, P_All_ = 0.02). Additionally, the TGF-beta pathway finding, which was more pronounced in non-hypermutated tumors (HR_NHM_ 1.30, 95% CI 1.10–1.50, *P* = 2.5 × 10^− 04^), remained statistically significant after adjustment for *SMAD4* (HR_NHM_ 1.25, 95% CI 1.04–1.51, P_NHM_ = 0.02) but not after adjustment for both *SMAD4* and *TGFBR2* (HR_NHM_ 1.20, 95% CI 0.99–1.46, P_NHM_ = 0.07).

### Simultaneous assessment of mutated gene combinations in survival analysis

To systematically investigate whether any combination of mutated genes was linked to survival we performed logic regression analyses. Using a 5-fold cross-validation, the optimal logic regression model was composed of two logic combinations (Fig. 1d). One logic combination was mutated *BRAF* p.V600E (tree 1), where patients with *BRAF* p.V600E mutations had worse survival outcomes than those without. The other logic combination was hypermutation status and mutated *ZFHX3* or MSI-H or mutated *B2M* (tree 2). In this model, patients with any of the following features were more likely to have favorable survival than those without these features: (1) hypermutated and *ZFHX3* mutation, (2) MSI-H, or (3) *B2M* mutation. The *ZFHX3* gene is frequently mutated in hypermutated tumors (42.2%, compared with 2.2% among non-hypermutated tumors), with a significant correlation between the two features (chi-square *P* < 2.2 × 10^− 16^). Comparing patients with the logic combination of tree 2 tumors to those without (i.e., MSS and non-mutated *B2M* with NMH or non-mutated *ZFHX3*), we observed improved DS-survival (HR 0.35, 95% CI 0.27–0.45, *P* < 2.0 × 10^− 16^). In the logic analyses stratified by hypermutation status, the optimal combination model for hypermutated-only tumors included one logic tree of mutated *HLA-B.* In the non-hypermutated-only subset, the best performing model included three logic trees of mutated *KRAS* (tree 1), *BRAF* p.V600E (tree 2), and *TP53* (tree 3).

## Discussion

We identified 9 novel significantly mutated genes and observed significant differences in somatic mutation prevalence by patient and tumor characteristics. These novel findings highlight additional biologically relevant drivers in colorectal tumors, broadening the catalog of genes implicated in colorectal cancer development. Additionally, mutations in *BRAF* p.V600E and several pathways were significantly associated with DS-survival, while other mutated genes were not significantly associated with survival after multiple-comparison correction.

The newly identified significantly mutated genes are involved in well-studied colorectal cancer pathways, transcriptional and post-transcriptional regulators, chromatin organization, and DNA damage and repair. Among these, *USP9X* emerged as a novel significantly mutated gene in colorectal cancer. *USP9X* encodes a deubiquitinating enzyme that regulates the TGF-beta signaling pathway by removing monoubiquitin from *SMAD4* at lysine 519, thereby restoring its ability to form active transcriptional complexes [19]. Loss of *USP9X* impairs TGF-beta signaling and promotes tumorigenesis. In addition, USP9X stabilizes the tumor suppressor FBW7, leading to degradation of oncogenic substrates such as MYC [20]. In our data, approximately 80% of *USP9X* variants are predicted to be damaging. These include loss-of-function changes (frameshift, stop-gain, splice-site, and non-frameshift deletions), as well as nonsynonymous variants predicted to be deleterious by REVEL, PolyPhen, or SIFT (Supplementary Table S5). When considering only non-hypermutated tumors, about 88% of variants identified in our data were classified as damaging (Supplementary Table S5 and Supplementary Figure S2). Further, reduced USP9X expression has been associated with poor prognosis in colorectal cancer, consistent with a tumor-suppressive role [20]. In our study, however, *USP9X* mutations were not associated with DS-survival. Collectively, these findings support *USP9X* as a biologically relevant and potentially functionally important regulator in colorectal cancer pathogenesis, warranting further investigation of its role in tumor progression and as a potential therapeutic target.

Several of the other newly identified significantly mutated genes converge on pathways fundamental to colorectal tumorigenesis, including transcriptional regulation, chromatin remodeling, and DNA damage response. While the present analysis is not intended to guide treatment selection, these findings highlight new candidates for future functional validation and integration with transcriptomic and epigenetic data in future studies to identify potential therapeutic targets in colorectal cancer. An oncoplot with REVEL, SIFT, and PolyPhen predictions is provided in Supplementary Information (Figure S2) to aid in evaluating potential functional effects for the nine novel significantly mutated genes in non-hypermutated tumors.

We also validated 7 of the genes recently identified as colorectal cancer driver genes, including two known drug targets, *IDH1* and *ERBB2* [21]. Specifically, we identified *IDH1* as being significantly mutated in non-hypermutated tumors. This gene was previously shown to impact brain tumors and the *IDH* mutation status is a prognostic marker [22]. In *IDH1*, the most prevalent mutations affected codon p.Arg132 (p.Arg132Cys/His/Leu), with mutations identified among 28 non-hypermutated tumors and 10 hypermutated tumors. The p.Arg132 substitutions abolish the enzyme’s ability to convert isocitrate to alpha-ketoglutarate, and instead acquires an enzymatic ability to convert alpha-ketoglutarate to 2-hydroxyglutarate [23]. The associations of *IDH1/2* mutations with older age and the *BRAF* p.V600E substitution have been described in colorectal cancer [24]. In our study, the *IDH1* p.Arg132 driver mutations were not associated with age or *BRAF* p.V600E variant allele frequency.

Tumors with *IDH1* p.Arg132 mutations have been shown to be treatable with drugs. For example, the IDH1-specific small-molecule inhibitor ivosidenib (p.Arg132 missense mutation), an approved drug for acute myeloid leukemias and cholangiocarcinomas, and vorasidenib were associated with a reduced tumor 2-hydroxyglutarate concentration, decreased tumor cell proliferation and immune activation in a phase 1 trial of low-grade glioma [25]. Furthermore, in a phase 1b/II clinical trial of *IDH1*-mutated myeloid cancers, combination therapy using ivosidenib, with or without the BCL2 inhibitors venetoclax and azacitidine, showed a promising effect for overcoming the resistance mechanism of a single IDH1 inhibitor [26]. If reproduced in the expansion phase of the trials, these treatments may apply to other IDH-mutated cancers, including colorectal cancer. These observations underscore the potential therapeutic relevance of IDH1 inhibition in colorectal cancer and align with a broader precision oncology framework in which metabolic driver alterations may represent druggable targets [25, 26]. However, the clinical efficacy of IDH1 inhibitors in colorectal cancer remains unproven and warrants further investigation.

Furthermore, we observed that *ERBB2* is significantly mutated in colorectal cancer. In clinical trials, the antibody-drug conjugates trastuzumab deruxtecan, trastuzumab plus pertuzumab, and zanidatamab (an antibody targeting two distinct ERBB2 (HER2) epitopes) have shown beneficial effects on *ERBB2*-positive (with a mutation and/or amplification) gastroesophageal junction cancer, breast cancer, lung cancer, biliary tract cancer, and RAS/BRAF wild type metastatic colorectal cancer [27–31]. The US Food and Drug Administration-approved anti-HER2 tucatinib plus trastuzumab treatment for chemotherapy-refractory ERBB2 (HER2)-positive RAS-wild type metastatic colorectal cancer has shown favorable outcomes [32]. Additionally, pyrotinib combined with trastuzumab has shown antitumor activity in ERBB2 (HER2)-positive RAS/BRAF wild-type advanced colorectal cancer [33]. Thus, patients with *ERBB2*-mutated colorectal tumors may also benefit from these treatments. The recognition of *HER2* (*ERBB2*) alterations as actionable targets has been reinforced by the phase II MOUNTAINEER trial of tucatinib plus trastuzumab and the DESTINY-CRC01/CRC02 studies of trastuzumab deruxtecan, which demonstrated durable responses and have led to inclusion of HER2-directed therapies in treatment guidelines for RAS/BRAF wild-type metastatic colorectal cancer [31, 32]. In our study, approximately 67% of *ERBB2* variants are predicted to be damaging. These include loss-of-function changes (frameshift, stop-gain, splice-site, and non-frameshift deletions), as well as nonsynonymous variants predicted to be deleterious by REVEL, PolyPhen, or SIFT (Supplementary Table S5).

We observed statistically significant differences in mutation prevalence by sex, tumor site and stage. *MXRA5*, located on Xp22.23, was more frequently mutated in tumors among women. This gene encodes for a secreted glycoprotein involved in matrix remodeling and cell adhesion and has been reported to be upregulated in several cancers, including colon cancer [34, 35]. Consistent with our findings, *BRAF* tumor mutations have been observed to be more prevalent among women [36, 37]. When accounting for MSI status and mutational burden, *BRAF*, *KRAS*, and *PIK3CA* mutations occurred more frequently in proximal tumors whereas *TP53* mutations occurred more frequently in distal tumors, consistent with previous studies [3, 7, 38, 39]. A review of *SMAD4*-mutated colorectal tumors similarly reported more frequent mutations among colon versus rectal tumors, but did not differentiate between proximal and distal colon [40]. Reported differences in mutation frequencies by tumor location for other genes have been inconsistent and may reflect differences in tumor location, approaches used to determine significance, and the inclusion of other clinicopathological factors, such as mutation burden and MSI status, in analyses. Observed differences in mutation prevalence across studies may also reflect variations in temporal contexts, such as changes in screening or treatment over time, population exposures and demographics, and sequencing technical or analysis pipelines. These factors can affect mutation detection, cohort composition, and tumor characteristics, complicating direct comparisons between studies. Limited prior research on some of the identified genes underscores the necessity for additional research to validate the observed differences in somatic mutation frequencies by tumor location. Finally, our observation of more frequent *BRAF* p.V600E mutations in later-stage tumors than in stage 1 tumors is consistent with prior reports and with our observation of an association with poorer DS-survival [39].

While later stage tumors had a greater frequency of *BRAF* p.V600E mutations than stage 1 tumors did, the association between *BRAF* p.V600E mutations and survival persisted with stage adjustment and was evident across case groups defined by tumor site and sex. The association between *BRAF* p.V600E mutation and colorectal cancer-specific death changed from 1.96 (*P* = 2 × 10^− 10^) to 1.70 (*P* = 5 × 10^− 06^) after adjusting for stage. This modest attenuation suggests that part of the effect of *BRAF* p.V600E on colorectal cancer-specific death may be due to its role in advancing the progression of cancer to a more advanced stage. In this context, stage is likely acting as a partial mediator of the relationship between a *BRAF* p.V600E mutation and DS-survival. Consistent with a growing number of studies demonstrating poorer outcomes among individuals with tumors exhibiting a combination of MSS and *BRAF*-mutated status, we observed that the association between *BRAF* mutation status and survival was limited to those with non-hypermutated tumors [41–45]. We observed similar results in our sub-analyses stratified by MSI and MSS status, with significantly poorer outcomes observed among individuals with MSS and *BRAF*-mutated tumors but not MSI and *BRAF*-mutated tumors (Supplementary Table S14). In contrast, *KRAS*-mutated tumors did not show differences in DS-survival by MSI versus MSS status, suggesting that the adverse survival effect is specific to *BRAF* rather than a shared feature of RTK/RAS pathway mutations (Supplementary Table S14). These findings, combined with our observation of a later stage at diagnosis among those *BRAF*-mutated tumors, may reflect the connection of *BRAF* somatic mutations to the serrated pathway of colorectal cancer etiology, which tends to be more aggressive and can emerge in flat, harder to detect precursor lesions [2]. Lastly, while the strong association between *BRAF* p.V600E mutations and colorectal cancer-specific death in female patients with rectal tumors is intriguing and may reflect underlying biological influences, the number of events in this subgroup is small. As such, these findings require validation in future studies.

Beyond single gene analyses, several pathways were significantly associated with DS-survival, consistent with previous studies [46]. Using an agnostic logic regression approach, we identified combinations of somatic features associated with DS-survival. Logic regression, unlike traditional (semi-) parametric models that evaluate single-gene effects, enables detection of combinatorial patterns among binary predictors and has been successfully applied to identify SNP-SNP interactions [47, 48]. In our analysis, the negative effect of *BRAF* p.V600E was consistent with results from the Cox model. Additionally, we identified that patients with *B2M* mutation or the combined hypermutation status and *ZFHX3* mutation exhibited more favorable DS-survival. The *B2M* mutation component aligns with our Cox model findings showing a marginal inverse association with DS-survival. Although *ZFHX3* mutations alone were not significantly associated with DS-survival, their occurrence within combinatorial patterns indicates potential biological relevance. *ZFHX3*, also called *ATBF1*, encodes a transcription factor with homeodomains and zinc finger motifs that suppress alpha-fetoprotein expression and negatively regulates MYB [49]. *A*cting as a tumor suppressor, *ZFHX3* has been implicated in prostate and breast cancers, and is frequently mutated in microsatellite unstable endometrial (36%) and colorectal (approximately 40%) tumors [11, 50–52]. *ZFHX3* loss promotes ESR1-mediated cell proliferation in ESR1-positive breast cancer cells by upregulating breast cancer stem cells and MYC transcription, and similar findings have been reported in androgen receptor-positive prostate cancer [50, 53]. Mutated *ZFHX3* is associated with favorable overall survival in non-small cell lung cancer patients who received immune checkpoint inhibitor treatments, and *ZFHX3* mutations are also positively correlated with known immunotherapy response biomarkers like neoantigen load, T cell infiltration, and higher tumor mutation load [54]. This evidence suggests that *ZFHX3* mutations may decrease colorectal cancer patients’ risk of DS-death, particularly in the context of hypermutation, through enhanced immune-related mechanisms. Our observed logic regression findings suggest that co-occurring genomic alterations may define tumor profiles impacting outcomes that are not apparent from single-gene analyses. These patterns warrant further investigation in molecularly and clinically annotated studies to assess their prognostic and biomarker potential. These findings also reinforce the clinical relevance of hypermutation and MSI status as predictive biomarkers for immunotherapy response, underscoring their continued importance in guiding colorectal cancer treatment. Lastly, while our logic regression analysis enabled identification of interpretable combinations of somatic features associated with DS-survival, other multivariable approaches, such as LASSO or Cox elastic net, could provide complementary insights and are worth exploring in future studies.

This study has several notable strengths, including its large sample size, the inclusion of population-based cases, and a targeted sequencing panel designed from prior whole exome sequencing studies, enabling deep coverage of established and putative colorectal cancer genes. However, some limitations should be acknowledged. The 199-gene panel necessarily excludes rare or emerging drivers that could be detected through exome- or genome-wide approaches. In addition, this analysis did not integrate transcriptomic, epigenetic, or immune profiling, which could provide additional functional context for somatic alterations. Findings from this population-based study highlight important avenues for future work, including integration of multi-omic data in sizable studies to investigate functional impacts and for replication in independent datasets.

Beyond population-based genomic discovery efforts, translating these findings toward clinical relevance will require complementary lines of investigation. Laboratory studies using experimental systems, such as in vitro, organoid or in vivo models, will be important for elucidating functional consequences of the identified mutations and clarifying their role in colorectal tumor biology. Analyses in independent, clinically annotated cohorts are needed to assess whether incorporating these somatic alterations improves risk stratification or prognostic performance beyond established clinicopathological factors. Examples from other malignancies, including validated prognostic calculators and EHR-integrated guideline support tools, illustrate the feasibility of such approaches. However, prospective evaluation within clinical studies will ultimately be needed to determine whether integrating genomic features into multivariable prognostic models or guideline-concordant electronic decision-support systems can meaningfully inform patient management in colorectal cancer.

## Conclusions

This sizable study offers valuable information about newly discovered significantly mutated genes, elucidates tumor heterogeneity with respect to patient and tumor characteristics, and reinforces prior research findings regarding the association between somatic mutations in *BRAF* and DS-survival while providing additional insights into this association with respect to patient sex and tumor site. Our observation that only a few mutated genes and combination of mutated genes impact DS-survival, while accounting for hypermutation status, suggests that most mutations driving colorectal tumor development have a limited impact on colorectal cancer survival.

## Supplementary Information

Supplementary Material 1.

Supplementary Material 2.

## Data Availability

Data Availability: The sequencing data from this study are available via the database of Genotypes and Phenotypes (dbGaP, https://dbgap.ncbi.nlm.nih.gov/ ) [55]. Accession numbers are phs001905 and phs002050.

## Abbreviations

CI: Confidence interval
CIDR: Center for Inherited Disease Research
CCFR: Colon Cancer Family Registry
CRC: Colorectal cancer
DEDD: Mutations in a POLD1 motif of highly conserved amino acids that bind magnesium (p.D316, p.E318, p.D402, p.D515)
DS-Survival: Disease specific survival
FFPE: Formalin-fixed paraffin-embedded
GECCO: Genetics and Epidemiology of Colorectal Cancer Consortium
HR: Hazard ratio
MSS: Microsatellite stable
MSI: Microsatellite instability
MSI-H: MSI-high
OICR: Ontario Institute for Cancer Research
OR: Odds ratio
TCGA: The Cancer Genome Atlas
