# Supplementary material for: Genomic characterization of colorectal tumors: insights into significantly mutated genes, pathways, and survival outcomes
Source: BMC Cancer. 2025 Dec 18;26:109. doi: 10.1186/s12885-025-15440-x (PMC12831426; doi:10.1186/s12885-025-15440-x)
Supplement: Supplementary file 1 — Supplementary Material 1. [file 12885_2025_15440_MOESM1_ESM.docx]

**Supplementary Information**

In the following we will describe methods details for all samples processed at the Center for Inherited Disease Research (CIDR). Details about samples processed at Ontario Institute for Cancer Research (OICR) have been described previously.^1^

**Study Descriptions**

**Cancer Risk Assessment (CRA)**^2^

All individuals who had surgery at the Mayo Clinic, Methodist Hospital, or St. Mary’s Hospital, Rochester, MN, who consented to participate from 1995 to 1998 were included in this study’s collection. Every patient was offered a chance to participate, no exclusion criteria were applied. Subjects who consented were given a form to complete that contained questions about lifestyle, medical history, and family history. Materials collected from subjects included peripheral blood, resected tumor from the center and the rim of the neoplasm, as well as normal colon both adjacent to the tumor and at the surgical margin. One representative piece from each was flash frozen and stored at -70C. The remaining piece was fixed in formalin and embedded in paraffin. Additionally, if lymph nodes or metastatic tumors were present, material was collected for these as well.

**Colorectal Cancer Family Registry (CCFR)**^3^

The CCFR is an NCI-supported consortium consisting of six centers dedicated to the establishment of a comprehensive collaborative infrastructure for interdisciplinary studies in the genetic epidemiology of colorectal cancer. The CCFR includes data from approximately 42,500 total subjects in 15,000 families (10,500 probands, and 26,770 unaffected and affected relatives and 4,276 unrelated controls and 923 spouse controls). Cases and controls, ages 20 to 74 years, were recruited at the six participating centers beginning in 1998. CCFR implemented a standardized questionnaire that is administered to all participants and includes established and suspected risk factors for colorectal cancer, which includes questions on medical history and medication use, reproductive history (for female participants), family history, physical activity, demographics, alcohol and tobacco use, and dietary factors. This study selected tumor samples from population-based cases in the Australian population-based center.

**Colorectal Cancer Genetics and Genomics (CRCGEN)**^4^

This Spanish study combines data from three case-control studies. The first one, performed in University Hospital of Bellvitge, L'Hospitalet, Barcelona, recruited incident pathology-confirmed CRC cases during the period 1996-1998. The second study was performed in the same hospital during the period 2007-2015 and the third study was conducted in Hospital of Leon, Leon, during 2008-2013. This study included both colorectal cancer cases and adenoma cases. Adenoma or serrated polyps were detected at screening colonoscopy. Patients with high-risk lesions were selected, defined as ≥ 5 adenomas/serrated polyps, or ≥ 1 adenoma/serrated polyp ≥ 20mm.

**European Prospective Investigation into Cancer and Nutrition, Norfolk site (EPIC-Norfolk)**^5^

The EPIC study is a multi-center, prospective cohort designed to investigate the associations between diet, cancer, and other chronic diseases across 10 European countries: Denmark, France, Germany, Greece, Italy, the Netherlands, Norway, Spain, Sweden and the United Kingdom (UK). Participants were recruited between 1992 and 1998 and included 521 330 men and women aged 35–70 years. Details of this study have been previously described and are available online (https://www.epic-norfolk.org.uk/). For this study, Formalin-fixed paraffin-embedded (FFPE) tumors from the EPIC Norfolk site were selected for inclusion. All colorectal cancers were diagnosed and treated at the Norfolk and Norwich Hospital, Norwich, UK.

**Health Professionals Follow-Up Study (HPFS)**^6–10^

The HPFS cohort comprises over 51,000 men aged 40-75 years at enrollment who were followed since the study started in 1986. Participants provided information on health-related exposures, including current and past smoking history, weight, height, diet, supplement use, alcohol intake, physical activity, aspirin use, endoscopy procedures, and family history of colorectal cancer every two years (or four years for diet) through questionnaires. Colorectal cancer and other outcomes were reported by participants or next-of-kin and were followed up through review of the medical and pathology record by physicians. Lethal unreported colorectal cancer cases were identified (and confirmed) through next-of-kin, use of the National Death Index and medical record review. Overall, more than 97% of self-reported colorectal cancers were confirmed by medical record review. Information was abstracted on histology, primary tumor location, TNM staging, tumor size and multiplicity, and the number of positive and negative lymph nodes. In 1993-1995, over 18,000 participants mailed blood samples by overnight courier, which were aliquoted into buffy coat and stored in liquid nitrogen. In 2001-2004, nearly 14,000 participants who had not provided a blood sample previously mailed in a swish-and-spit sample of buccal cells. FFPE tissue blocks were collected from hospitals where participants with colorectal carcinoma had undergone tumor resection or endoscopic biopsy (for pre-operatively treated rectal cancer). The study pathologist reviewed hematoxylin-and-eosin (H&E)-stained tissue sections and recorded histopathological features.

**Hispanic Colorectal Cancer Study (HCCS)**^11^

HCCS is a population-based study of individuals self-identified as Hispanic with a diagnosis of colorectal cancer. Cases are identified from the California Cancer Registry or directly from local hospitals in the Los Angeles region [LAC + USC County Hospital and University of Southern California Norris Comprehensive Cancer Center]. All men and women over 21 years of age with a first-time diagnosis of CRC (ICD-O-3 codes: C18–C21) after January 1, 2008, were eligible for participation. Risk factor and dietary questionnaires, pathology reports, and saliva samples (for genotyping) were collected using methodologies developed in the Colon Cancer Family Registry and the Multiethnic Cohort. Participants recruited into the HCCS were born in Mexico, the US, Central/South America, Cuba, the Caribbean Islands, or Europe. The present study includes cases with FFPE colorectal tumor tissue available.

**Iowa Women’s Health Study (IWHS)**^12,13^

In the IWHS, a 16-page baseline questionnaire was completed and returned by 41,836 randomly selected women, ages 55 to 69 years, who resided in Iowa and held a valid driver’s license at baseline in 1986. Comprehensive self-reported demographic, dietary, lifestyle, and medication data were collected during the baseline IWHS evaluation (1986). Incident colorectal cancer cases were identified through annual linkage with the Iowa Cancer Registry, which is a member of the National Cancer Institute’s Surveillance, Epidemiology, and End Results (SEER) program. Colorectal cancer cases were identified using International Classification for Diseases in Oncology (ICD-O) codes. Beginning in 2006, archived, paraffin-embedded tissue specimens were requested from incident colorectal cancer cases diagnosed through December 31, 2002.

**Melbourne Collaborative Cohort Study (MCCS)**^14^

The MCCS is a prospective cohort study of 41,513 healthy adult volunteers between the ages of 27 and 76 years (99% aged 40-69) recruited from the Melbourne metropolitan area between 1990 and 1994. By 31 December 2009, 1,046 participants had a first histopathological diagnosis of invasive adenocarcinoma of the colon or rectum identified by a record linkage to the Victorian Cancer Registry following the baseline study visit. Beginning in 2004, archived formalin-fixed paraffin embedded archived tissue specimens were requested from incident colorectal cancer cases diagnosed through 1995 to 2009. All CRC cases eligible for this study were selected based on the availability of a tumor sample and having no pre-baseline history of CRC (as confirmed by the Victorian Cancer Registry).

**Nurse’s Health Study (NHS)**^8–10,15^

The NHS cohort began in 1976 when over 121,000 female registered nurses ages 30 to 55 years returned the initial questionnaire that ascertained baseline characteristics. Participants provided information on health-related exposures, including current and past smoking history, weight, height, diet, supplement use, alcohol intake, physical activity, aspirin use, endoscopy procedures, and family history of colorectal cancer every two years (or four years for diet) through questionnaires. Colorectal cancer and other outcomes were reported by participants or next-of-kin and were followed up through review of the medical and pathology record by physicians. Lethal unreported colorectal cancer cases were identified (and confirmed) through next-of-kin, use of the National Death Index and medical record review. Overall, more than 97% of self-reported colorectal cancers were confirmed by medical record review. Information was abstracted on histology, primary tumor location, TNM staging, tumor size and multiplicity, and the number of positive and negative lymph nodes. FFPE tissue blocks were collected from hospitals where participants with colorectal carcinoma had undergone tumor resection or endoscopic biopsy (for pre-operatively treated rectal cancer). The study pathologist reviewed H&E-stained tissue sections and recorded histopathological features.

**Nurse’s Health Study II (NHS-II)**^16^

The Nurses' Health Study II (NHS-II) is an ongoing cohort of over 116,000 female registered nurses in the US, aged 25-42 years at baseline in 1989. Participants provided information on health-related exposures, including current and past smoking history, weight, height, diet, supplement use, alcohol intake, physical activity, aspirin use, endoscopy procedures, and family history of colorectal cancer every two years (or four years for diet) through questionnaires. Colorectal cancer and other outcomes were reported by participants or next-of-kin and were followed up through review of the medical and pathology record by physicians. Lethal unreported colorectal cancer cases were identified (and confirmed) through next-of-kin, use of the National Death Index and medical record review. Overall, more than 97% of self-reported colorectal cancers were confirmed by medical record review. Information was abstracted on histology, primary tumor location, TNM staging, tumor size and multiplicity, and the number of positive and negative lymph nodes. Study participants who had not previously reported a diagnosis of cancer and had responded to the 1995 study questionnaire were invited to provide blood samples between 1996 and 1999. Blood samples were collected from over 29,000 participants, aged 32 to 54 years at the time of blood draw. Similarly, between 2004 and 2006, active study participants who had not previously provided a blood sample were invited to provide buccal samples. Swish-and-spit samples of buccal cells were received from nearly 30,000 participants. FFPE tissue blocks were collected from hospitals where participants with colorectal carcinoma had undergone tumor resection or endoscopic biopsy (for preoperatively treated rectal cancer). The study pathologist (S.O.) reviewed H&E-stained tissue sections and recorded histopathological features.

**Prostate, Lung, Colorectal, and Ovarian Cancer Trial (PLCO)**^17,18^

PLCO is a large, randomized, two-arm trial that enrolled over 154,000 men and women between the age of 55 and 74 years at ten centers across the U.S. to evaluate the effectiveness of screening on cancer mortality for malignancies arising in the prostate, lungs, ovaries, colon and rectum. Half of the participants were randomized into the screening arm and half into the control arm. Participants in the screening arm received sigmoidoscopy screening at baseline and year 3 or 5 of the trial; participants in the control arm received usual care. Enrollment began in 1993 and concluded in 2001. Both arms were followed for cancer incidence and mortality for at least 13 years from baseline. Details of this study have been previously described and are available online (http://dcp.cancer.gov/plco). In 2006 through 2012, FFPE pathology tissue samples were collected from PLCO participants who developed a selected cancer, including colorectal cancer. This study includes cases with sufficient material for sequencing.

**Women’s Health Initiative (WHI)**^19^
The WHI study is a large, multi-center study of postmenopausal women aged 50 to 79 years at recruitment from 40 US clinical centers between 1993 and 1998, including over 68,000 women who participated in four overlapping trials evaluating: menopausal hormone therapy (HT: two trials), dietary modification (DM) and calcium-vitamin D (CaD) supplementation. Participants in the CaD trial were recruited from those who were either in the HT or the DM trial. Details of the WHI study design have been described elsewhere and are available online (https://www.whi.org/). FFPE pathology tissue samples were collected from WHI participants who developed selected cancers, including colorectal cancer. Patients with sufficient material and consent were included in this study.

**Targeted Sequencing**

**Targeted sequencing panel genomic content**

To ensure the most comprehensive selection of putative relevant genes and other somatic mutations related to colorectal cancer (CRC) we primarily selected genes based on 1,211 whole exome sequencing paired normal-tumor samples including CRC patients with formalin-fixed paraffin embedded (FFPE) tissue from the HPFS and NHS, as well as fresh frozen tissue from The Cancer Genome Atlas (TCGA) colon and rectal adenocarcinoma data.^20–22^ These datasets were separately called and analyzed for somatic mutations. Analyses were conducted stratified by cases with hypermutated samples (defined as > 17 SNVs/Mb) and non-hypermutated samples. A primary inclusion criterion for selecting a gene was that the gene was significantly mutated (p < 0.05 based on MutSigCV analysis) in non-hypermutated cases in either the TCGA samples or in the HPFS/NHS samples.

Furthermore, we conducted pathway analysis based on the results from MutSigCV to identify CRC-related pathways and additional genes within relevant pathways. We conducted nonrandom cluster analysis to identify additional genes with the gain of function mutations not identified otherwise. We evaluated the list of significantly mutated genes for the hypermutated samples based on HPFS/NHS and TCGA. However, as the list was very long and likely included false positive findings, we only included a gene if additional evidence was available, such as a low p-value in non-hypermutated samples, support from the literature review, or presence in highlighted pathways. In addition to these individual level data analyses, we conducted a literature search, evaluated genes listed in the Catalogue of Somatic Mutations in Cancer (COSMIC, http://cancer.sanger.ac.uk/cosmic) and the TumorPortal CRC datasets (http://www.tumorportal.org), and added genes recommended by consortium (GECCO) collaborators.^1,20,23–32^ These approaches led to the selection of 298 genes. As MSI testing is possible with the next generation sequencing we included 236 MSI/homopolymer markers. As a quality control measure, we included a gender marker. Genomic regions with focal copy number alterations in the TCGA-CRC dataset were also included.^20^ The list of genomic regions is presented in Supplementary Table S15.

**Library preparation, sequencing, and processing**

We performed a low input library prep protocol developed by our team.^33^ Libraries were prepared from 50-200 ng of genomic DNA, sheared for 80 seconds using the Covaris LE220plus instrument (Covaris). The Kapa Hyper prep kit was used to process the sheared DNA into amplified dual-indexed adapter-ligated fragments. All processing was done in 96 well plate formats using robotics (Beckman FXp, Perkin Elmer Janus, Agilent Bravo, Beckman NX). ‘With Bead’ clean-ups were used following shearing and adapter ligation using GE Healthcare Sera-Mag Magnetic SpeedBeads (Carboxylate-Modified) beads. A total of 750 ng of the amplified library was used in a pooled enrichment reaction (8 samples per normal pool, 4 samples per tumor pool) following IDT protocols (4-hour hybridization, Integrated DNA Technologies). Post-capture amplification was performed using the Kapa HiFi PCR enzyme with custom primers. Libraries were sequenced on the NovaSeq 6000 platform using 47 bp paired-end runs and sequencing chemistry kits NovaSeq 6000 S2 Reagent Kit. NovaSeq flowcell data were demultiplexed using Picard (picard-version 2.17.6). ExtractIlluminaBarcodes followed by IlluminaBasecallsToSam. Output from IlluminaBasecallsToSam was read group level BAM files encoding the Unique Molecular Identifier (UMI) sequence and quality score in RX and QX tags respectively. Samples that were run in multiple flow cell lanes (multiple read groups) had all read group level BAM files merged into a single BAM file using picard-2.17.6 MergeSamFiles. Unaligned reads in CRAM format with UMI were aligned to GRCh37/hg19 reference genome using Burrows-Wheeler Aligner (BWA-MEM version 0.7.17). Mapped and tagged reads were collapsed into consensus reads following the fgbio workflow (Fulcrum Genomics-version 1.2.0). Molecular consensus reads were locally realigned and used for downstream analysis.

**Sequencing data quality control (QC)**

To assess the quality of sequencing data, we examined quality metrics using Picard 2.18.29 (http://broadinstitute.github.io/picard) (Supplementary Table S16). We generated CollectHSMetrics on the consensus read libraries sequenced and processed. Output metrics were used to calculate percent target bases at 20x coverage or higher. To pass the read depth quality threshold, at least 80% of target reads of both the primary tumor and matched normal needed to be above 20x coverage. Samples that failed to reach sufficient coverage and libraries were selected for re-sequencing.

All samples that passed coverage QC with pre-existing genotyping data from GWAS (n=1,001) were checked for single nucleotide polymorphism (SNP) concordance. Germline SNPs were called with HaplotypeCaller from Genome Analysis Toolkit (GATK 4.0.1.1) with option “max_alternate_alleles” set to 3 for all target intervals to generate likelihoods that the sites were homozygous reference or not.^34^ Germline variant filtering was performed on the joint called VCF using the following cut-offs from fields contained in the INFO column: SNPs - QD < 2.0, MQ < 40.0, FS > 60.0, MQRankSum < -12.5 and ReadPosRankSum < -8.0; and InDels and mixed variants - QD < 2.0; FS > 200.0 and ReadPosRankSum < -20.0. An additional VCF file was created where genotypes for biallelic SNPs were further refined using CalculateGenotypePosteriors using allele frequency information from 1000 genomes phase 3 data as well as Exome Aggregation Consortium data (r0.3).

Additional hybrid-selection metrics of interest were Read pass filter aligned ratio (PCT_PF_READS), AT dropout (AT_DROPOUT), and GC dropout (GC_DROPOUT). The Median insert size for each tumor library was calculated using CollectInsertSizeMetrics. Chimeric pairs ratio was determined from the tool CollectAlignmentSummaryMetrics (PCT_CHIMERAS).

**Somatic mutation calling and filtering***Point mutations*

Somatic point mutations were called from samples that passed minimum coverage criteria for every tumor and matched normal samples using Strelka2 (version 2.9.9) and Mutect2 (GATK, version 4.1.4.1).^35,36^ Strelka2’s somatic workflow was run with matched tumor-normal BAM files and the hg19 reference genome. Mutect2’s workflow was run with our in-*house* panel of normals (PoN) generated from matched normal samples as additional germline and sequencing artifacts filter. The PoN was created in 3 steps: 1. Run MuTect2 on matched normal samples with adjacent normal tissue samples excluded, 2. Filter MuTect2 to a minimum variant allele frequency of 5%, and 3. Combine VCF files of passed calls.

*Short insertions or deletions (InDels)*

Short somatic InDels (< 50 base pairs in length) were called from matched tumor-normal pairs in a two-step process. First, we used Manta (version 1.6.0) to create the first list of candidates. Manta was configured to run in exome mode to switch off high-depth filters. Second, we ran Strelka2 with Manta’s list of candidates using the targeted/exome mode. We also called InDels with Mutect2, which called and filtered InDels simultaneously with point mutations.

*Mutation candidate annotation and filtering*

We annotated calls with ANNOVAR to predict functional consequences and the structural context of somatic mutations.^37^ We also configured ANNOVAR to annotate variants with information from dbSNP, COSMIC, and ClinVar databases.^38,39^ We filtered Mutect2 calls with Mutect2’s read orientation artifacts workflow to reduce the deamination effects of formalin fixation on sequencing (https://gatk.broadinstitute.org/hc/en-us/articles/360035531132--How-to-Call-somatic-mutations-using-GATK4-Mutect2). Followed by applying MuTect2’s standard filter suite (https://github.com/broadinstitute/gatk/blob/master/docs/mutect/mutect.pdf). We limited candidate point mutations and InDels to those that passed the filter status in Strelka2 and Mutect2. We left-aligned and took the intersection of mutation calls from Strelka2 and MuTect2 and further applied the following customized filters. A mutation was dropped if any of the following criteria were met:

- CONTQ < 93; CONTQ: Phred-scaled quality that alternative allele is not due to contamination.
- SEQQ < 93; SEQQ: Phred-scaled quality that alternative allele is not due to sequencing error.
- *abs*(MPOS - 12) < 6; MPOS: Relative median distance of point mutation to end of read (*abs* denotes the absolute value).
- STRANDQ <= 40; STRANDQ: Phred-scaled quality of point mutation not being a strand bias artifact.
- SSEVS < 10.5 and AF < 0.2; SSEV: Strelka2’s somatic empirical variant scoring and AF denotes the allelic frequency in the tumor (Mutect2).
- exac03nontcga > 0.0001: Minor allele frequency in population from Exome Aggregation Consortium (ExAC) database (excluding TCGA).
- InDels with AF < 0.2; AF denotes allelic frequency in the tumor (Mutect2).

*Significantly mutated genes*

After data QC, and mutations and indel calling, we used data on all mutations for application of MutSigCV.^40^ We utilized a likelihood ratio test to identify genes significantly mutated beyond the background mutation rate (Supplementary Figures S1 and S2).

**
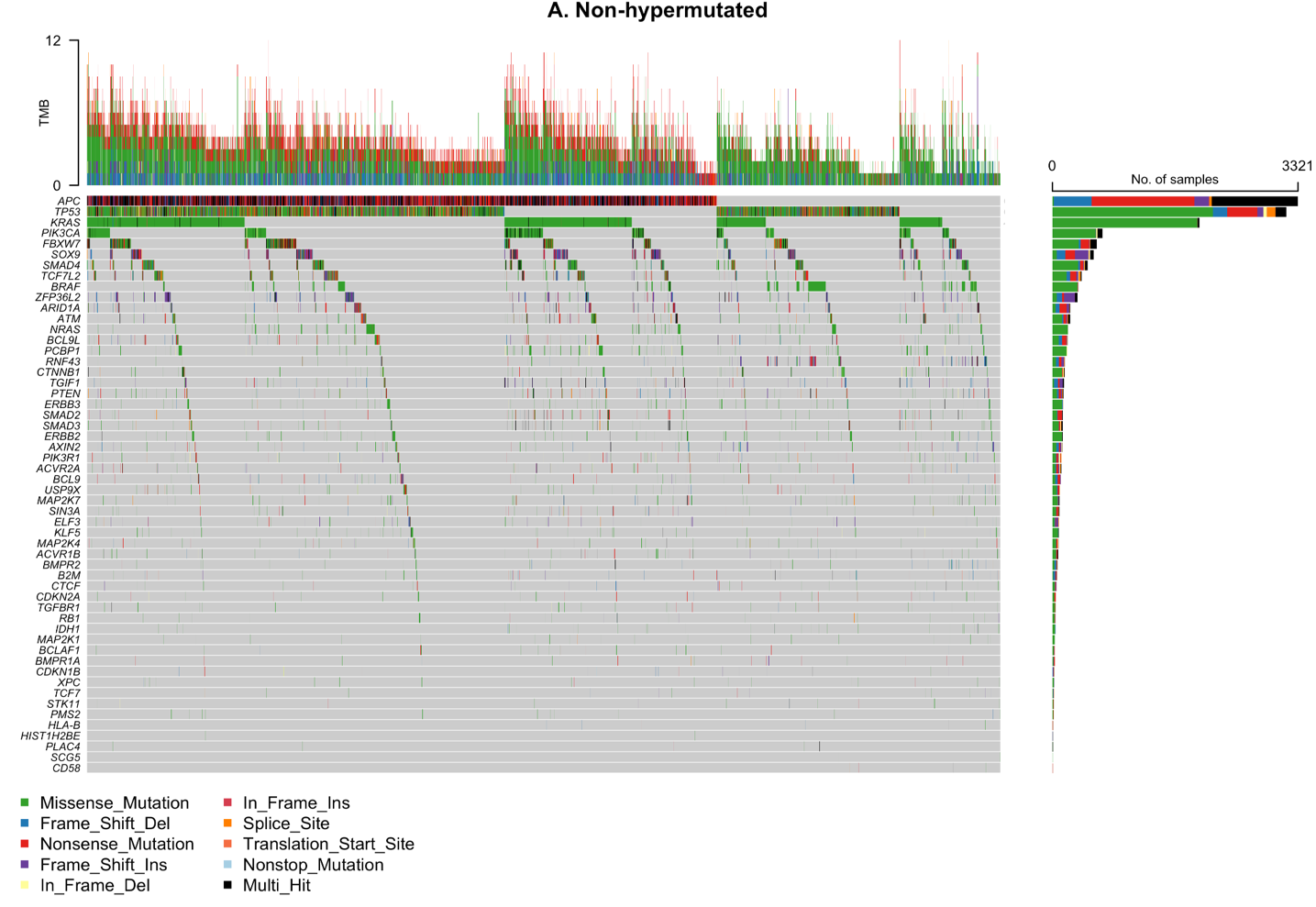
**

**
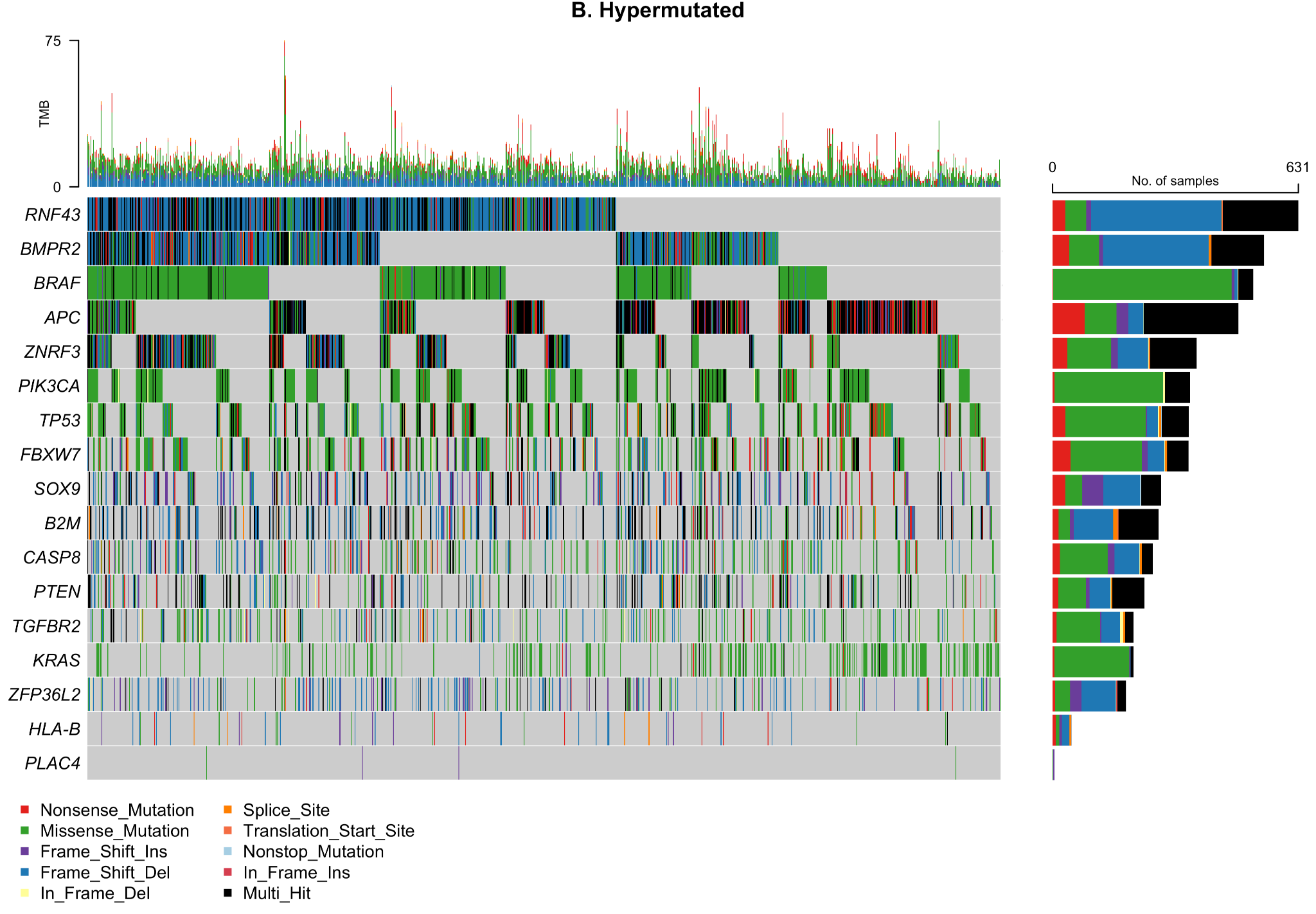
Supplementary Figure S1. Oncoplots of all significantly mutated genes for hypermutated and non-hypermutated tumors**. Top panels show the number of mutations detected in each sample. The right panels display the number of variants per gene. A) Non-hypermutated tumors. B) Hypermutated tumors.

**
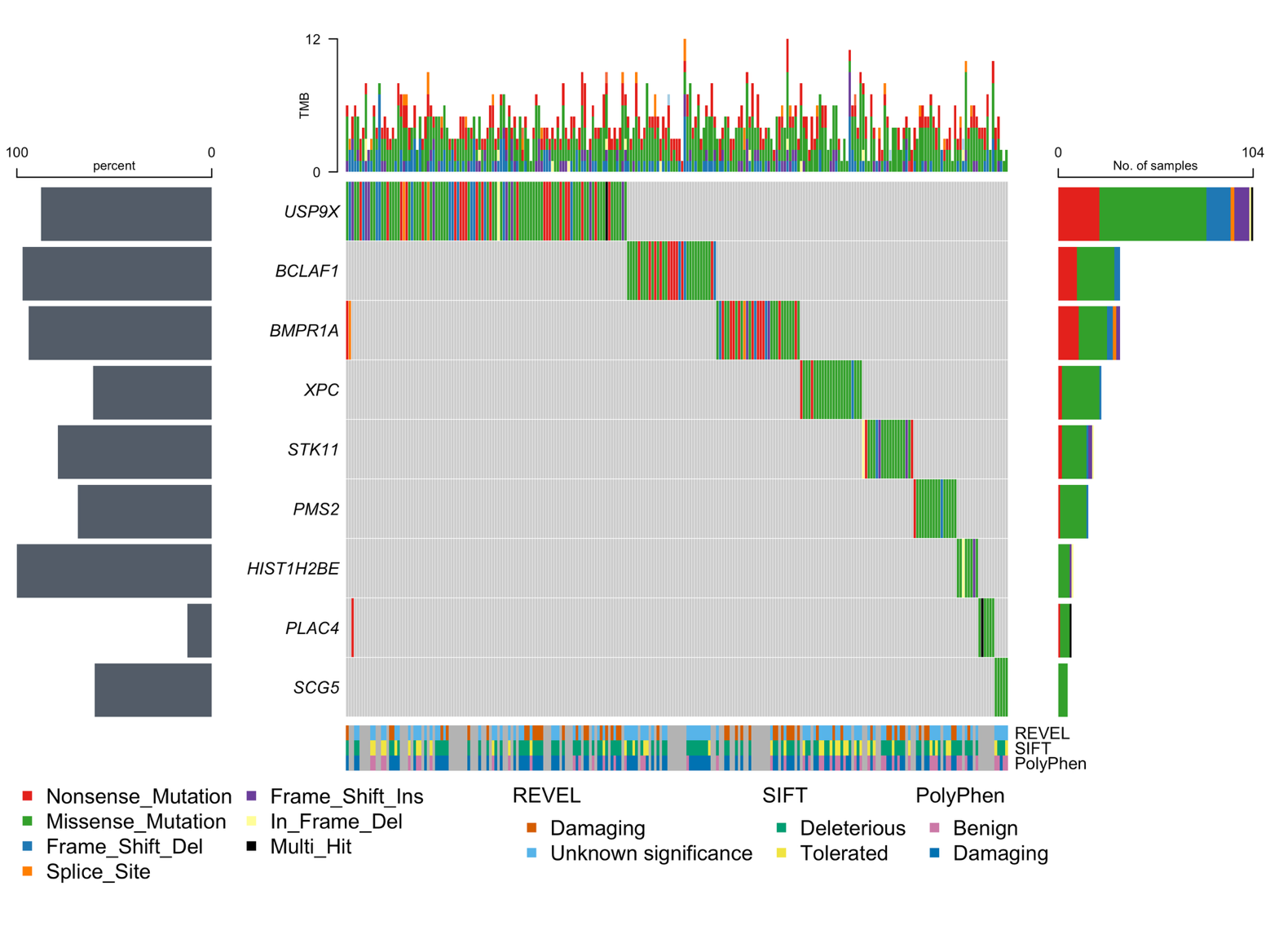
Supplementary Figure S2. Oncoplot of the novel significantly mutated genes in non-hypermutated tumors with annotations**. Top panels show the number of mutations detected in each sample. The right panel displays the number of variants per gene. The left panel is the percent of variants per gene that are nonsense, frameshift, splice site, nonstop, translational start site, or damaging missense mutations (defined as a missense mutation with a damaging or deleterious REVEL, SIFT, or PolyPhen annotation). The bottom panel shows the most damaging variant REVEL, SIFT, and PolyPhen annotations.

**Post-mutation calling QC**

To further improve data calls, we used five tumor-normal sample pairs that were sequenced in our previous targeted sequencing project.^1^ Additionally, we attempted to validate 85 point mutations in the new dataset using pyrosequencing. Of the 85 mutations, 28 pyrosequencing assays failed or only a tumor or normal samples was successfully sequenced. Therefore, we excluded these 28 point mutations. Further, two point mutations were false positive for the pyrosequencing. One for which the mutation in *CDK12* was adjacent to an InDel, causing poor primer performance, and one for which the pyrosequencing primer was inside a homopolymer in *MAP2K7*. This left 55 remaining point mutations successfully sequenced in the validation set. All remaining 55 point mutations validated and, accordingly, validation with an orthogonal method showed 100% concordance of the point mutations between our targeted sequencing and pyrosequencing.

We also used pyrosequencing to evaluate the quality of the InDels. Of the 36 InDels, 12 assays failed or only the tumor or normal sample was successfully sequenced - these 12 were excluded. An additional InDel was a false positive for the pyrosequencing, as the pyrosequencing ended in a homopolymer in *SOX9*. All remaining 20 InDels validated. Accordingly, validation showed 100% concordance of InDels between our targeted sequencing and pyrosequencing.

Furthermore, we selected 100 mutations (61 point mutations and 39 InDels) from the EPIC-Norfolk cohort for Sanger sequencing. Three sequencing runs repeatedly failed and were dropped. One point mutation could not be validated and is likely a false positive. The remaining 96 were successfully validated showing 98.9% concordance -100% of InDels and 98.3% of point mutations.

To estimate the number of potential false-negative results, we selected a third method of validation, Agena iPlex. We designed iPlex genotyping panels for 2 x 36 sites in 96 tumor samples (i.e., 6,912 reactions testing 1,248 deletions, 288 insertions and 5,376 SNVs). A total of 177 reactions failed and had to be dropped from final assessment. A deletion in the *APC* gene at locus chr5:112175951 showed an unusually high false-negative rate and we performed 22 Sanger sequencing. Sanger sequencing confirmed a skewed cluster from MALDI-TOF spectrometry analysis and that targeted sequencing calls were correct. In total, of the 6,735 sites tested, 6,719 confirmed genotypes from targeted sequencing. In total, 16 sites were discordant, 12 deletions and 4 SNVs resulting in overall 99.7% concordance (99% for deletions, 100% insertions and 99.9% SNVs).

**Somatic mutation definitions**

*List of 199 genes*

*ABCA8, ACVR1B, ACVR2A, AKT1, ALK, AMER1, APC_NM_000038_truncated_first1600AA, ARID1A, ARID1B, ARID2, ARID3A, ASXL1, ATG2A, ATM, ATXN1, AXIN1, AXIN2, B2M, BCL9, BCL9L, BCLAF1, BCOR, BIRC6, BMPR1A, BMPR2, BRAF_NM_004333_V600, C11ORF53, CACNG3, CASP8, CCDC13, CCDC40, CD274, CD58, CDC27, CDH1, CDK12, CDKN1B, CDKN2A, CHD4, COLCA2, CSNK2A1, CTCF, CTNNB1_NM_001098209_hotspot, CTNND1, CUX1, DAPK1, DCAF4L1, DCC, DCHS1, DNMT1, DPYD, DUSP16, DYNC1H1, ELF3, ELMO1, ENAM, EP300, EP400, EPCAM, ERBB2, ERBB3, ERCC3, ESR1, FAM171B, FAN1, FAT1, FBLN2, FBXW7, FETUB, FGFR1, GDF5, GNAS, GPC5, GREM1, HCN1, HGF, HIST1H2BE, HLA.B, IDH1, IDH2, IGF2, IWS1, KDM6A, KIF1A, KLF5, KLHL11, KLHL5, KMT2B, KMT2C, KMT2D, KRAS_NM_033360_Oncogenic_known, LIMCH1, LMO7, LRRN3, MAML2, MAP2K1, MAP2K4, MAP2K7, MAST2, MLH1, MLH3, MLLT3, MSH2, MSH6, MTUS2, MUC4, MUTYH, MXRA5, MYC, MYH9, N4BP2L1, NCAPD3, NCF4, NFE2L3, NLGN4X, NRAS_NM_002524_Oncogenic_known, NRG1, NTHL1, OSBPL6, PAX3, PAX5, PBRM1, PCBP1, PCDH10, PCDHA3, PCDHGA7, PCDHGA9, PCDHGB1, PDCD1LG2, PIK3CA_NM_006218_nonsynonymous_SNV, PIK3R1_NM_181523_non_silent, PLAC4, PMS2, POLD1, POLE, PPP2R1A, PRKCI, PTEN_NM_000314_non_silent, RB1, RBM10, RECQL5, RGMB, RGS12, RNF43, RNF43_NM_001305544_truncating, RYR1, S1PR4, SALL4, SCG5, SCN5A, SETD2, SIN3A, SLC12A5, SMAD2, SMAD3, SMAD4, SMARCA4, SMG1, SOS1, SOX9, SPZ1, STK11, SYNE1, SYT3, TAF1L, TAF3, TBX3, TCF7, TCF7L2, TCHH, TET1, TET2, TET3, TEX14, TGFBR1, TGFBR2, TGIF1, TNRC6B, TP53_NM_000546_non_silent, TPR, TRIP4, TSHZ2, TYRO3, URI1, USP9X, UTP20, XPC, XPO6, ZBTB7A, ZFHX3, ZFP36L2, ZHX2, ZNF217, ZNF493, ZNF521, ZNF678, ZNF681, ZNF99, ZNRF3*

*Mutated variants and pathways*

We annotated variants using ANNOVAR, defining gene mutations based on the presence of non-silent mutations in an exonic region. An SNV was considered non-silent if it was non-synonymous, stop-gain, stop-loss, or splicing variant, and an indel was considered silencing if it was a frameshift deletion, frameshift insertion, in-frame deletion, in-frame insertion, stop-gain, or stop-loss. We refined this definition for a subset of genes with known functional effects of mutations (*BRAF*: Codon V600E mutations, *CTNNB1* hotspot, *KRAS*: codons G12, G13, Q61, K117, A146 mutations, *PIK3CA*: Transcript NM181523, and *TP53*: Transcript NM00546 encoding for the canonical p53). We defined six primary gene sets implicated in CRC as being mutated if any gene within that set was mutated. These set included: TP53/ATM; receptor tyrosine kinases/RAS (RTK/RAS) genes (*BRAF, ERBB2, ERBB3, KRAS, NRAS*), transforming growth factor (TGF-beta) genes (*ACVR1B, ACVR2A, BMPR1A, BMPR2, GDF5, SMAD2, SMAD3, SMAD4, TGFBR1*, and *TGFBR2*), IGF2/phosphatidylinositide 3-kinases (PI3K) genes (*IGF2, PIK3CA, PIK3R1, and RNF43*), the WNT/beta-catenin (CTNNB1) signaling pathway (*AMER1, APC, ARID1A, AXIN1, AXIN2, CTNNB1, FBXW7, SOX9, TCF7, TCF7L2*), and mismatch repair (MMR) genes (*MLH1, MLH3, MSH2, MSH6*, and *PMS2*).

*MSI and hypermutation status*

We called microsatellite instability (MSI) using MSIsensor2, defining tumors with a score >18.0% as MSI-high (MSI-H) and microsatellite stable (MSS) otherwise. We empirically determined a mutation burden threshold to define hypermutation status, defining tumors with >26 somatic mutations (13 mutations per million bases) as hypermutated and non-hypermutated otherwise (Supplementary Figure S2).


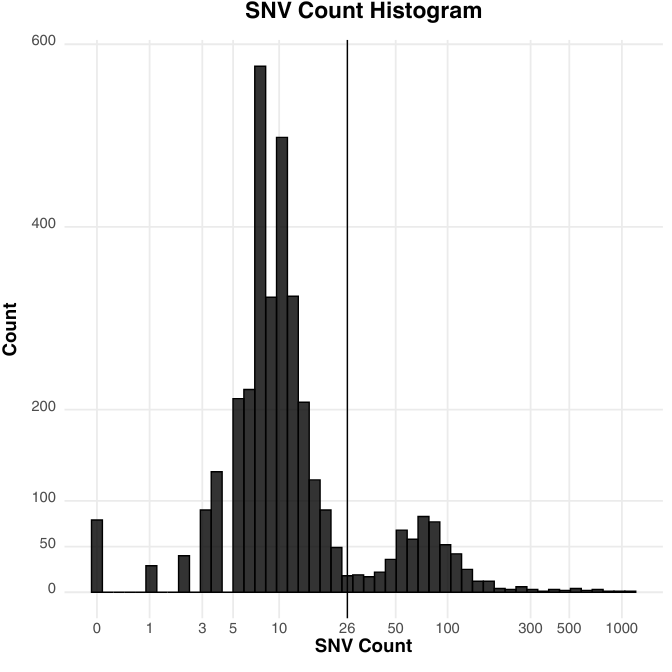


**Supplementary Figure S3. Histogram of point mutations (single nucleotide variants, SNVs) in the log-scale.** The threshold to define a hypermutated tumor was set to 26 mutations.

*In silico annotations of variant calls for significantly mutated* genes

We used the Ensembl Variant Effect Predictor (VEP; release 115, cache/build GRCh37) to annotate variants in significantly mutated genes.^41^ For each variant, we report the following prediction metrics in Supplementary Table S5: REVEL (Rare Exome Variant Ensemble Learner), PolyPhen prediction and score, SIFT (Sorting Intolerant from Tolerant) prediction and score, CADD (Combined Annotation Dependent Depletion) PHRED score, MPC (Missense badness), ClinPred, and the VEP IMPACT category.^41-47^ These *in silico* predictors are provided to prioritize potentially deleterious alterations.

**Supplementary Information References**

1. Zaidi SH, Harrison TA, Phipps AI, et al. Landscape of somatic single nucleotide variants and indels in colorectal cancer and impact on survival. Nat Commun. 2020;11(1):3644. doi:10.1038/s41467-020-17386-z

2. Cunningham JM, Kim CY, Christensen ER, et al. The frequency of hereditary defective mismatch repair in a prospective series of unselected colorectal carcinomas. Am J Hum Genet. 2001;69(4):780-790. doi:10.1086/323658

3. Newcomb PA, Baron J, Cotterchio M, et al. Colon Cancer Family Registry: an international resource for studies of the genetic epidemiology of colon cancer. Cancer Epidemiol Biomarkers Prev. 2007;16(11):2331-2343. doi:10.1158/1055-9965.EPI-07-0648

4. Obón-Santacana M, Díez-Villanueva A, Alonso MH, et al. Polygenic risk score across distinct colorectal cancer screening outcomes: from premalignant polyps to colorectal cancer. BMC Med. 2021;19(1):261. doi:10.1186/s12916-021-02134-x

5. Riboli E, Hunt KJ, Slimani N, et al. European Prospective Investigation into Cancer and Nutrition (EPIC): study populations and data collection. Public Health Nutr. 2002;5(6B):1113-1124. doi:10.1079/PHN2002394

6. Giovannucci E, Liu Y, Platz EA, Stampfer MJ, Willett WC. Risk factors for prostate cancer incidence and progression in the health professionals follow-up study. Int J Cancer. 2007;121(7):1571-1578. doi:10.1002/ijc.22788

7. Haruki K, Kosumi K, Li P, et al. An integrated analysis of lymphocytic reaction, tumour molecular characteristics and patient survival in colorectal cancer. Br J Cancer. 2020;122(9):1367-1377. doi:10.1038/s41416-020-0780-3

8. Nishihara R, Wu K, Lochhead P, et al. Long-term colorectal-cancer incidence and mortality after lower endoscopy. N Engl J Med. 2013;369(12):1095-1105. doi:10.1056/NEJMoa1301969

9. Mehta RS, Nishihara R, Cao Y, et al. Association of Dietary Patterns With Risk of Colorectal Cancer Subtypes Classified by Fusobacterium nucleatum in Tumor Tissue. JAMA Oncol. 2017;3(7):921-927. doi:10.1001/jamaoncol.2016.6374

10. Wang L, He X, Ugai T, et al. Risk factors and incidence of colorectal cancer according to major molecular subtypes. JNCI Cancer Spectr. 2021;5(1). doi:10.1093/jncics/pkaa089

11. Schmit SL, Schumacher FR, Edlund CK, et al. Genome-wide association study of colorectal cancer in Hispanics. Carcinogenesis. 2016;37(6):547-556. doi:10.1093/carcin/bgw046

12. Folsom AR, Kaye SA, Prineas RJ, Potter JD, Gapstur SM, Wallace RB. Increased incidence of carcinoma of the breast associated with abdominal adiposity in postmenopausal women. Am J Epidemiol. 1990;131(5):794-803. doi:10.1093/oxfordjournals.aje.a115570

13. Tillmans LS, Vierkant RA, Wang AH, et al. Associations between cigarette smoking, hormone therapy, and folate intake with incident colorectal cancer by TP53 protein expression level in a population-based cohort of older women. Cancer Epidemiol Biomarkers Prev. 2014;23(2):350-355. doi:10.1158/1055-9965.EPI-13-0780

14. Giles GG, English DR. The Melbourne Collaborative Cohort Study. IARC Sci Publ. 2002;156:69-70.

15. Belanger CF, Hennekens CH, Rosner B, Speizer FE. The nurses’ health study. Am J Nurs. 1978;78(6):1039-1040.

16. Bao Y, Bertoia ML, Lenart EB, et al. Origin, methods, and evolution of the three nurses’ health studies. Am J Public Health. 2016;106(9):1573-1581. doi:10.2105/AJPH.2016.303338

17. Zhu CS, Huang W-Y, Pinsky PF, et al. The prostate, lung, colorectal and ovarian cancer (PLCO) screening trial pathology tissue resource. Cancer Epidemiol Biomarkers Prev. 2016;25(12):1635-1642. doi:10.1158/1055-9965.EPI-16-0506

18. Black A, Huang W-Y, Wright P, et al. PLCO: evolution of an epidemiologic resource and opportunities for future studies. Rev Recent Clin Trials. 2015;10(3):238-245. doi:10.2174/157488711003150928130654

19. Hays J, Hunt JR, Hubbell FA, et al. The Women’s Health Initiative recruitment methods and results. Ann Epidemiol. 2003;13(9 Suppl):S18-77. doi:10.1016/S1047-2797(03)00042-5

20. Grasso CS, Giannakis M, Wells DK, et al. Genetic mechanisms of immune evasion in colorectal cancer. Cancer Discov. 2018;8(6):730-749. doi:10.1158/2159-8290.CD-17-1327

21. Cancer Genome Atlas Network. Comprehensive molecular characterization of human colon and rectal cancer. Nature. 2012;487(7407):330-337. doi:10.1038/nature11252

22. Giannakis M, Mu XJ, Shukla SA, et al. Genomic Correlates of Immune-Cell Infiltrates in Colorectal Carcinoma. Cell Rep. 2016;15(4):857-865. doi:10.1016/j.celrep.2016.03.075

23. Bellido F, Pineda M, Aiza G, et al. POLE and POLD1 mutations in 529 kindred with familial colorectal cancer and/or polyposis: review of reported cases and recommendations for genetic testing and surveillance. Genet Med. 2016;18(4):325-332. doi:10.1038/gim.2015.75

24. Kandoth C, McLellan MD, Vandin F, et al. Mutational landscape and significance across 12 major cancer types. Nature. 2013;502(7471):333-339. doi:10.1038/nature12634

25. Guda K, Moinova H, He J, et al. Inactivating germ-line and somatic mutations in polypeptide N-acetylgalactosaminyltransferase 12 in human colon cancers. Proc Natl Acad Sci USA. 2009;106(31):12921-12925. doi:10.1073/pnas.0901454106

26. Ngeow J, Heald B, Rybicki LA, et al. Prevalence of germline PTEN, BMPR1A, SMAD4, STK11, and ENG mutations in patients with moderate-load colorectal polyps. Gastroenterology. 2013;144(7):1402-1409, 1409.e1. doi:10.1053/j.gastro.2013.02.001

27. Lawrence MS, Stojanov P, Mermel CH, et al. Discovery and saturation analysis of cancer genes across 21 tumour types. Nature. 2014;505(7484):495-501. doi:10.1038/nature12912

28. Yang H, Gao Y, Feng T, Jin T-B, Kang L-L, Chen C. Meta-analysis of the rs4779584 polymorphism and colorectal cancer risk. PLoS ONE. 2014;9(2):e89736. doi:10.1371/journal.pone.0089736

29. Tuupanen S, Hänninen UA, Kondelin J, et al. Identification of 33 candidate oncogenes by screening for base-specific mutations. Br J Cancer. 2014;111(8):1657-1662. doi:10.1038/bjc.2014.429

30. Nahorski MS, Lim DHK, Martin L, et al. Investigation of the Birt-Hogg-Dube tumour suppressor gene (FLCN) in familial and sporadic colorectal cancer. J Med Genet. 2010;47(6):385-390. doi:10.1136/jmg.2009.073304

31. Fabregat A, Jupe S, Matthews L, et al. The Reactome Pathway Knowledgebase. Nucleic Acids Res. 2018;46(D1):D649-D655. doi:10.1093/nar/gkx1132

32. Rohlin A, Eiengård F, Lundstam U, et al. GREM1 and POLE variants in hereditary colorectal cancer syndromes. Genes Chromosomes Cancer. 2016;55(1):95-106. doi:10.1002/gcc.22314

33. Marosy BA, Craig BD, Hetrick KN, et al. Generating Exome Enriched Sequencing Libraries from Formalin-Fixed, Paraffin-Embedded Tissue DNA for Next-Generation Sequencing. Curr Protoc Hum Genet. 2017;92:18.10.1-18.10.25. doi:10.1002/cphg.27

34. van der Auwera G, O’Connor BD. Genomics in the Cloud: Using Docker, GATK, and WDL in Terra. (O’Reilly Media, ed.).; 2020.

35. Kim S, Scheffler K, Halpern AL, et al. Strelka2: fast and accurate calling of germline and somatic variants. Nat Methods. 2018;15(8):591-594. doi:10.1038/s41592-018-0051-x

36. Cibulskis K, Lawrence MS, Carter SL, et al. Sensitive detection of somatic point mutations in impure and heterogeneous cancer samples. Nat Biotechnol. 2013;31(3):213-219. doi:10.1038/nbt.2514

37. Wang K, Li M, Hakonarson H. ANNOVAR: functional annotation of genetic variants from high-throughput sequencing data. Nucleic Acids Res. 2010;38(16):e164. doi:10.1093/nar/gkq603

38. Tate JG, Bamford S, Jubb HC, et al. COSMIC: the catalogue of somatic mutations in cancer. Nucleic Acids Res. 2019;47(D1):D941-D947. doi:10.1093/nar/gky1015

39. Sherry ST, Ward M, Sirotkin K. dbSNP-database for single nucleotide polymorphisms and other classes of minor genetic variation. Genome Res. 1999;9(8):677-679. doi:10.1101/gr.9.8.677

40. Lawrence MS, Stojanov P, Polak P, et al. Mutational heterogeneity in cancer and the search for new cancer-associated genes. Nature. 2013;499(7457):214-218. doi:10.1038/nature12213

41. McLaren W, Gil L, Hunt SE, et al. The Ensembl Variant Effect Predictor. *Genome Biology*, 2016;17(1):122. doi:10.1186/s13059-016-0974-4.

42. Ioannidis NM*, Rothstein JH*, Pejaver V, et al., REVEL: An ensemble method for predicting the pathogenicity of rare missense variants. *Am J Hum Genet*, 2016;99(4):877-885. doi: 10.1016/j.ajhg.2016.08.016.

43. Adzhubei IA, Schmidt S, Peshkin L, et al., A method and server for predicting damaging missense mutations. *Nat Methods*, 2010;7(4):248-249. doi: 10.1038/nmeth0410-248.

44. Ng PC, Henikoff S. SIFT: Predicting amino acid changes that affect protein function. *Nucleic Acids Res*. 2003;31(13):3812-4. doi: 10.1093/nar/gkg509.

45. Kircher M, Witten DM, Jain P, et al., A general framework for estimating the relative pathogenicity of human genetic variants. *Nat Genet* 2014;46(3):310-5. doi: 10.1038/ng.2892.

46. Samocha KE, Kosmicki JA, Karczewski KJ, et al. Regional missense constraint improves variant deleteriousness prediction. bioRxiv. 2017. doi: 10.1101/148353.

47. Alirezaie N, Kernohan KD, Hartley T, et al., ClinPred: Prediction tool to identify disease-relevant nonsynonymous single-nucleotide variants. *Am J Hum Genet* 2018;103(4):474-83. doi: 10.1016/j.ajhg.2018.08.005.
